# Avian Influenza Viruses: Global Panzootic, Host Range Expansion and Emerging One-Health Threats

**DOI:** 10.3390/vetsci13010067

**Published:** 2026-01-09

**Authors:** Luigi Bruno, Maria Anna Nappo, Raffaele Frontoso, Salvatore Montinaro, Rosanna Di Lecce, Chiara Guarnieri, Luca Ferrari, Attilio Corradi

**Affiliations:** 1 Department of Prevention, Azienda Sanitaria Locale (A.S.L.) Napoli 3 Sud, 80053 Castellammare di Stabia, Italy; m.nappo@aslnapoli3sud.it; 2 Istituto Zooprofilattico Sperimentale del Mezzogiorno (I.Z.S.M.), 80055 Portici, Italy; 3 Azienda Socio-Sanitaria Locale (A.S.L.), 07100 Sassari, Italy; salvatore.montinaro@aslsassari.it; 4 Department of Veterinary Science, University of Parma, 43126 Parma, Italy; rosanna.dilecce@unipr.it (R.D.L.); chiara.guarnieri1@unipr.it (C.G.); attilio.corradi@unipr.it (A.C.)

**Keywords:** avian influenza virus (AIV), zoonosis, host range expansion, cell tropism, immune response, ecosystem, One-Health, legislation

## Abstract

The review deals with the current knowledge on the global panzootic spread of highly pathogenic avian influenza viruses (HPAIVs), with an emphasis on the H5N1 clade 2.3.4.4b virus. It describes the viral structure, replication, pathotypes and molecular determinants of host range, including sialic-acid receptor usage and key genetic mammalian-adaptation markers (PB2-E627K and PB2-D701N mutations). The host spectrum nowadays extends from wild waterfowl and poultry including seabirds, terrestrial and marine mammals and, based largely on experimental studies or molecular detection, reptiles, amphibians, and fish. Recently, the H5N1 clade 2.3.4.4b virus has shown marked tropism for lactating mammary epithelium in dairy cattle, with virions shed in raw milk. The review reports epidemiology, geographical expansion, clinical presentation, pathogenesis and pathology, diagnosis, immune responses and vaccination approaches across species. It also analyses European Union (EU) and Italian regulatory frameworks, surveillance strategies and biosecurity measures from a One-Health perspective. The review highlights how climate change, wildlife–livestock interfaces, intensive farming and global trade favor viral persistence and genomic reassortment and concludes by stressing strategic actions to limit further host adaptation and panzootic/pandemic risks.

## Simple Summary

The global spread of highly pathogenic avian influenza viruses (HPAIVs) is a major One-Health concern. HPAIVs infect a wide range of hosts, including wild birds, poultry, marine and semi-aquatic mammals, amphibians, fish, companion animals and equids. Recently, the H5N1 clade 2.3.4.4b virus has shown tropism for mammary epithelial cells in lactating dairy cattle, and the virions showed the ability to spread in raw milk. Climate change, wildlife–livestock interfaces and intensive farming may favor viral persistence and genomic reassortment. Coordinated action, grounded in national and international sanitary regulations and integrating surveillance, vaccination, biosecurity and environmental monitoring, is strategic and essential to limit further host adaptation as well as panzootic and pandemic risks.

## 1. Introduction

Avian influenza viruses (AIVs) belong to the species Alphainfluenzavirus, genus Alphainfluenzavirus, family Orthomyxoviridae. AIVs are highly contagious respiratory viruses of birds, causing high morbidity and mortality depending on the specific strain. AIVs are enveloped negative-sense ssRNA viruses that comprise 8 genomic segments encoding at least 11 proteins, either structural or non-structural. The polymerase complex, composed of the polymerase basic protein 1 (PB1), PB2, and polymerase acidic protein (PA), together with the nucleoprotein (NP), matrix protein 1 (M1), non-structural protein 1 (NS1), and nuclear export protein (NEP or NS2), is located within the lipid envelope. In contrast, the membrane ion channel (M2), hemagglutinin (HA), and neuraminidase (NA) are in the envelope. The molecular properties of HA and NA determine the classification of IVs into subtypes. To date, 19 HA (H1–H19) and 11 NA (N1–N11) subtypes have been identified, with the H17N10 and H18N11 “flu-like” subtypes being isolated exclusively in bats. Wild aquatic birds are considered the natural reservoirs for 17 out of the 19 HA (H1–16 and H19) and 9 out of the 11 NA (N1–9) subtypes, typically carrying influenza infection in a subclinical form [1].

In birds, classification ranges from H1 to H16 and N1 to N9. Lineage A of highly pathogenic avian influenza viruses (HPAIVs) is considered as the major global threat among HPAIVs. Many viruses possess zoonotic potential, with a significant impact on human health. Depending on the virus–host interaction, these viruses can alter their epidemiological impact. Low pathogenic avian influenza viruses (LPAIVs) belonging to the H5 and H7 subtypes may evolve into HPAIVs when infecting poultry, causing severe clinical signs and high mortality rates, particularly in members of the order *Galliformes* [2]. To date, 16 HA subtypes and 9 NA subtypes have been identified in both domestic and wild birds. IVs carrying the H5 subtype have been circulating in wild birds and domestic poultry since 1995 [3]. Wild aquatic birds belonging to the order *Anseriformes* (mainly ducks, geese, and swans) and *Charadriiformes* (gulls, terns, and shorebirds) are the natural reservoir of LPAIVs. From wild birds, LPAIVs can be transmitted directly or indirectly to domestic birds, other wild and domestic animals, and humans [4]. LPAIVs can cause diseases ranging from mild to severe in poultry farms (“spillover”). From poultry, the viruses can infect (“spillback”) wild birds again, and viruses may circulate asymptomatically or cause disease and mortality [5].

The evolution and epidemiology of the viruses depend on the interaction between the virus, the host, and the environment. For both LPAIVs and HPAIVs, an important component of viral evolution and epidemiology occurs at the interface between wild and domestic environments [6]. The pathogenicity of AIVs is based on lethality, clinical signs, and molecular characteristics. LPAI is a mild disease, often unnoticed or asymptomatic. In contrast, HPAI is a severe systemic disease, showing high morbidity and mortality. Until now, the geographic distribution of AI based on its pathogenic lineages has not been fully understood. However, from 1880 to 1959, AI spread among birds across Europe, Asia, Africa, and North and South America. HPAI caused the mass death and depopulation of approximately 316 million poultry between 2005 and 2021, affecting more than 50 countries. Outbreaks have led to the culling of about 2.5 million poultry worldwide, with the H5N1 subtype predominating. AI is a global public health concern due to its zoonotic potential. Additionally, outbreaks have been reported in free-ranging and captive wild mammals [7,8,9,10,11].

The most recognized zoonotic AIV is the H5N1 subtype. Globally, human cases have been reported with the following subtypes: H5N1 (870 cases and 430 deaths), H7N9 (1500 cases and 600 deaths), H5N6 (80 cases and 30 deaths), and H9N2 (80 cases and 2 deaths). Sporadic cases have been attributed to the subtypes H3N8, H7N4, H7N7, and H10N3. From 2003 to 2022, 868 human cases of H5N1 viral infection with 457 deaths were reported to the Pan-American Health Organization/World Health Organization (PAHO/WHO) from 21 different countries. Several human cases of AI were reported in 2022 [12]. HPAI is widespread across all continents through movements of both domestic poultry and transmission among/migration of wild avian species.

The evolution of lineages in recent years has resulted in geographic expansion and an increase in the species where the virus is detected, including spillover events and extension of the host range to mammalians in different environments. Until its emergence in the late 1990s, the Guangdong lineage of the HPAIV H5 was recognized as a threat to both the poultry industry and human health, largely due to limited immunity against this subtype and the clinical phenotype [13]. AI represents a significant global public health challenge due to its spread and circulation and high mortality [14]. Humans were affected by influenza pandemics caused by H1N1 in 1918, H2N2 in 1957–1958, H3N2 in 1968, and H1N1 (H1N1pdm09) virus in 2009, resulting in millions of deaths and enormous economic losses. The deadliest influenza pandemic in 1918 spread to one-third of the world’s population—approximately 500 million people—and caused at least 50 million deaths. Population movements across different regions of the planet, including remote areas, and increased travel speed, facilitated by technological advances, are aggravating factors which were not present in 1918 and could allow for faster global spread of dangerous viruses. The 1918 influenza pandemic, known as the “Spanish flu”, was the deadliest in history, with an estimated mortality rate of 2.5% or higher. It uniquely caused deaths among young adults, characterized by a “cytokine storm” and secondary bacterial pneumonia [15].

In recent years, the transmission of HPAI strains, especially H5N1, H5N8, and H7N9 viruses, has posed a significant public health threat. Among these, H5N1 virus is potentially the most pathogenic, with high mortality in both poultry and humans. From 2003 to 2023, the WHO has documented 878 human cases of HPAIV H5N1 infection, resulting in 458 deaths (52%) across 23 countries [14]. Recently, several regions worldwide have reported cases of HPAIV H5N1 infection, raising concerns among global health authorities. The current HPAIV H5N1 derives from the original A/goose/Guangdong/1/96 strain [16]. This infection, observed in the U.S. and other countries, represents the largest outbreak among wild birds [9,17]. Between 2022 and 2023, with data collected up to March 2023, numerous outbreaks of HPAIV H5N1 were reported in over 28 European countries, affecting various species of wild birds, poultry, and domestic birds [14]. The impact on the marine ecosystem was also notable. A potential transmission between pelicans and sea lions was hypothesized, highlighting complex viral transmission dynamics among different mammalian species. Such widespread dissemination among bird populations may facilitate virus persistence and increase the risk of transmission to other species, including those of economic importance, and humans [14]. Although sustained transmission among mammals has not been documented yet, the persistent recirculation in wild and domestic mammalian populations is concerning due to the potential viral adaptation and inter-species spillovers. Barriers to inter-species adaptation include changes within the viral polymerase complex to achieve efficient replication in mammalian cells and enhanced receptor-binding activity of HA and NA. The conformation of HA is particularly important as it mediates binding to the host cell receptor through a receptor binding site (RBS) and initiates the viral replication cycle [1,18]. Since 2021, the HPAIV H5N1 clade 2.3.4.4b virus has spread globally, causing morbidity and mortality in domestic poultry and impacting the poultry product supply chain and human food safety [16,18].

This review was undertaken to address existing gaps by providing a comprehensive synthesis of data on epidemiology, distinctive clinical and pathological features, diagnostic methodologies, and immunology of the disease. A structured literature search was performed in the PubMed and Google Scholar databases, employing predefined search terms to retrieve publications issued from 2005 to 2025. Some data reported in the tables of the AIV pathogenesis chapter derive from studies prior to 2005, as such unique studies are deemed essential to ensure the scientific completeness of the topic.

The importance of novel events and issues such as H5N1 infection in cattle, milk transmission potential, and cross-species genetic adaptation markers (PB2-E627K and PB2-D701N mutations) is underlined.

Furthermore, the review provides an integrated overview of the current regulatory framework for disease surveillance and control, encompassing national legislation in Italy and European Union (EU) directives, in agreement with the One-Health approach that promotes collaboration across veterinary, public, and environmental health sectors. Nevertheless, important challenges persist, particularly with respect to wildlife surveillance, early detection of cross-species spillover events, and preparedness for unconventional transmission routes such as exposure to raw milk.

## 2. Viral Cycle

The viral genome comprises 8 RNA segments highly prone to mutation and recombination, able to produce new viral variants which can bypass pre-existing host immunity [17]. Both point mutations in critical epitopes, referred to as “antigenic drift” and “antigenic shift” resulting from the reassortment of viruses originating from two or more hosts infecting a common host, can generate a novel, life-threatening virus that escapes recognition by antibodies previously developed in humans, swine, and domestic fowl populations, thereby conferring pandemic potential [19,20].

The life cycle of HPAIVs starts when a bird becomes infected through either direct or indirect exposure to feces, respiratory secretions, or contaminated environment. After entry, the virus undergoes primary replication within the epithelial cells of the host’s respiratory and gastrointestinal tracts.

The complete life cycle can be outlined as follows:

(1) primary infection: transmission occurs through contact with infectious biological fluids or ingestion of contaminated material;
(2) viral replication: active multiplication takes place within the respiratory and gastro-intestinal mucosae, resulting in the shedding of large numbers of virions;
(3) systemic dissemination: hematogenous spread to internal organs; typical clinical signs include depression, multi-organ hemorrhages, and acute death;
(4) environmental excretion: high viral loads are released into the environment through feces and respiratory secretions, thus promoting further transmission.

The conformation of HA plays a crucial role as it mediates the attachment to the host cell receptor through a specific receptor-binding site (RBS) and triggers the onset of the viral replication cycle. AIVs generally exhibit a preference for α2,3-linked sialylated glycans, which are predominantly distributed in the respiratory and gastrointestinal tracts of avian species, whereas mammalian IVs preferentially recognize α2,6-linked sialylated glycans, mainly found in mammalian tissues. Following endocytic uptake, the acidification of the endosome induces a conformational rearrangement in HA, facilitating the fusion of the viral envelope with the endosomal membrane. This event enables the release of the viral genome into the cytoplasm. HA is initially produced as an inactive precursor (HA0) that requires a proteolytic cleavage at a specific site to generate HA1 and HA2 subunits becoming functional and capable of driving membrane fusion and genome release. The amino acid composition at the cleavage site determines the spectrum of host enzymes able to process and activate HA. Most LPAI strains possess a single Arginine residue (monobasic) at the HA cleavage site, which is cleaved extracellularly by proteases secreted in the pulmonary and intestinal epithelium, thereby restricting infection to localized tissues and resulting in milder disease. Conversely, when the cleavage site contains multiple basic amino acids (polybasic cleavage site), it can be processed by different serine proteases, allowing for viral replication in a broad range of cell types and leading to a systemic and often fatal infection. Consequently, the cleavage site sequence is used by the WHO as a reference criterion for classifying AIVs by “pathotype” [1].

This property allows HPAIVs to replicate across a broad spectrum of cell types and tissues, increasing their capacity for systemic dissemination in terrestrial poultry. A distinctive feature of HPAIV pathogenesis in infected terrestrial birds is the pronounced infection of endothelial cells. This tropism is evident in multiple organs, including the lungs, heart, liver, and brain. Endothelial cells constitute the interface between the cardiovascular system and adjacent tissues while also maintaining vascular homeostasis, modulating coagulation pathways, and coordinating immune responses. Consequently, viral infections that induce endothelial cell dysfunction or loss often result in severe systemic outcomes, characterized by hemorrhages, edema, and coagulopathy. HPAIVs in wild birds do not exhibit endothelio-tropism, irrespective of the disease severity, and the viral antigen predominantly localizes in the parenchyma rather than within the vasculature [21].

HA harbors the major antigenic determinants targeted by the host’s humoral immune response, which is subjected to substantial selective pressure. The accumulation of non-synonymous nucleotide substitutions may enable the virus to evade neutralization by antibodies. More extensive genetic alterations can further influence viral characteristics, including receptor affinity, replication efficiency, and transmissibility [22]. In addition, the segmented nature of the AIV genome facilitates antigenic shifts. In avian species, such reassortment events among distinct AIV lineages induced the emergence of several novel zoonotic subtypes containing internal gene cassettes derived from other circulating strains. This mechanism also contributed to the origin of major human influenza pandemics [1].

## 3. Global Dynamics and Host Range Expansion

The natural ecological niche of AIVs consists of aquatic birds encompassing over 100 wild bird species across approximately 25 different families, indicating the global distribution of the virus in free-ranging aquatic birds [23]. AIV subtypes (H5, H7, H6, and H9) are found in aquatic birds, poultry, and in different mammals. AI subtypes in equines (H3 and H7), in swine (H1 and H3), and in aquatic mammals (H10, H4, H7, and H13) originate from the intrinsic genetics of viruses naturally present in wild aquatic birds (H1–H16) [12].

**Figure 1:**
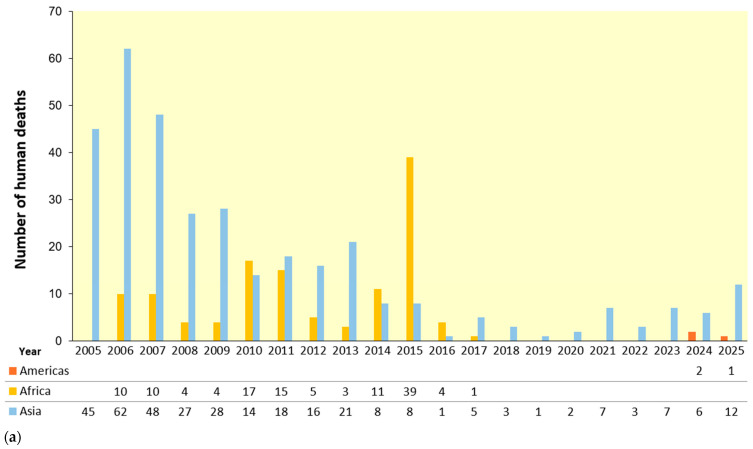

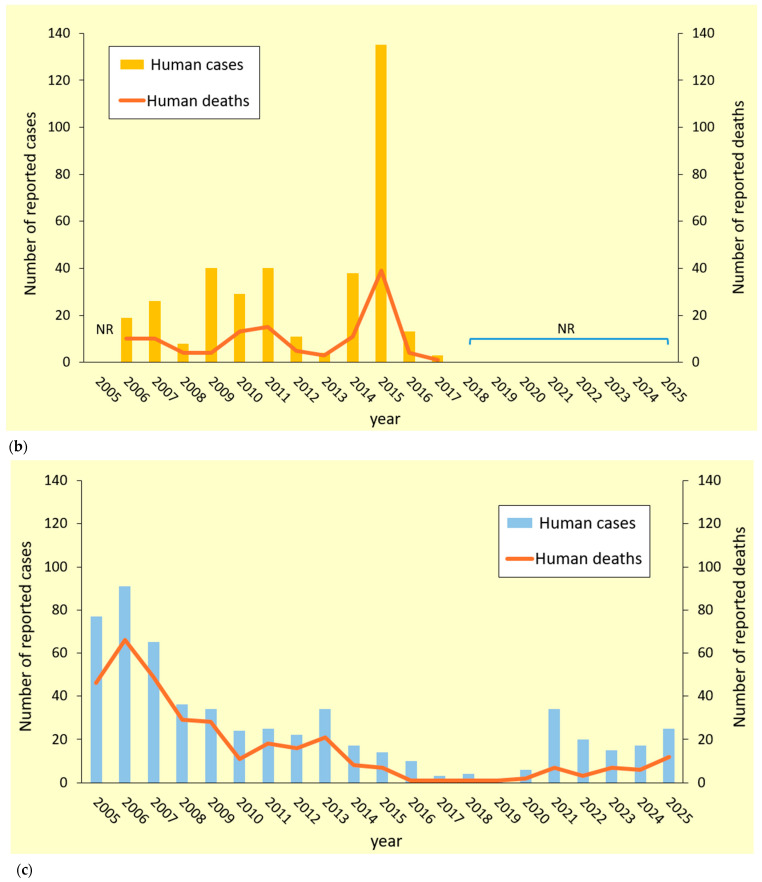
(**a**) Reported human deaths related to highly pathogenic avian influenza virus (HPAIV) H5 infections in different countries in the time-frame 2005–2025; (**b**) number of reported cases of human H5 virus infection in relation with the number of human H5 virus-related deaths in the same year in Africa; NR: not reported data (in 2005 and in 2017–2025); (**c**) number of reported cases of human H5 virus infection in relation with the number of human H5 virus-related deaths in the same year in Asia (data source: EMPRES-i+ Global Animal Disease Information System; latest access on 5 January 2026).

The shift toward a persistent panzootic phase became evident following the 2015 North American outbreak, which presented a critical turning point in the global epidemiology of HPAIVs. More than 50 million poultry were culled following the spread of Asian H5N8 viruses, which reassorted with North American LPAIV to generate novel H5N2 and H5N8 genotypes [24,25,26]. This event demonstrated that long-distance migratory flyways serve as effective routes for viral transmission, and intensive farming systems amplify the spread, potentially leading to severe outbreaks. Between 2016 and 2019, the global situation was influenced by the ongoing reassortment of clade 2.3.4.4 viruses. H5N8 and H5N6 subtypes were prevalent in Asia, Europe, and Africa and caused recurring poultry losses and occasional wild bird die-offs [27,28]. This period was characterized by the rise in the H5N6 virus as a zoonotic pathogen in China, where several confirmed human cases presented severe pneumonia and a case fatality rate (CFR) exceeding 40% [29] (Figure 1).

Surveillance in Europe and Africa showed that the genetic plasticity of H5Nx viruses enables them to persist in wild birds as reservoirs, and migratory waterfowl serve as maintenance hosts [30].

The epidemiological scenario changed significantly after 2020 with the detection of the H5N1 clade 2.3.4.4b virus. Beyond its temporal significance, this phase was characterized by a marked expansion of the host range, with increasing involvement of wild birds and mammalian species [31,32]. In Europe, repeated infections were reported each winter, and high mortality events in barnacle geese and swans highlighted the virus pathogenicity in previously resilient wild hosts [33]. In West and North Africa, infection in poultry highlighted the vulnerability in regions with limited veterinary infrastructures [34,35]. By 2021, the virus also become widespread in North America, where surveillance identified infection in migratory ducks, geese, and raptors [36]. The global diffusion reached South America in 2022, marking an epidemiological milestone that highlighted the virus ability to infiltrate entirely new ecosystems. The spread of infections by the H5N1 clade 2.3.4.4b virus along the Pacific and Atlantic coasts caused unprecedented mortality in marine mammals, particularly in South American sea lions (*Otaria flavescens*) in Peru, Chile, and Argentina [37,38]. These mortality events, involving thousands of animals, were consistent with epidemiological and phylogenetic patterns that suggest potential mammal-to-mammal transmission, particularly under high-density colony conditions. Almost simultaneously, the virus was found in Antarctica, where brown skuas and penguins tested positive [39,40]. These detections marked the first entry of HPAIVs into the Antarctic ecosystem, raising concerns of irreversible ecological disruption in naive seabird populations.

HPAIV H5N1 has the ability to infect both animals and humans. Although transmission of HPAIV H5N1 from birds to humans is considered rare and human-to-human transmission is infrequent, a limited number of human cases with high mortality rates have been documented from multiple countries since 2003. Considering the changes observed in the transmission pattern of HPAIV H5N1 during 2022–2023 in Europe, enhanced surveillance of zoonotic transmission events between animals and humans is recommended. Before 2005, significant transmission of HPAIV H5N1 from poultry to wild birds was relatively rare. However, a shift has occurred, marking a new phase in the epidemiological dynamics of HPAIV H5N1 in wild birds. The rapid and concerning spread of HPAIV H5N1 among domestic and wild bird populations is a transcontinental phenomenon [14]. HPAIV H5N1 has been naturally isolated from a wide range of other animal species, including cats, dogs, foxes, seals, leopards, mustelids (mink and otters), badgers, tigers, lions, pikas, martens, porpoises, raccoons, raccoon dogs, pigs, Virginia opossums, civets, bears, dolphins, stone/marten foxes, coyotes, and even fish, among others [41].

AIVs, after reaching the intestinal tract of wild birds, can be shed through saliva, feces, and nasal secretions. Human infection with HPAIV H5N1 mainly occurs through direct contact with infected birds. Movement of HPAIV H5N1 in airborne particles covers distances extending less than 10 m [42,43]. Importantly, the detection of viral RNA in airborne particles should not be interpreted as direct evidence of infectious virus or effective airborne transmission. However, within larger particles containing viral RNA, environmental dispersion has been reported at distances of up to approximately 80 m; however, the infectivity of the virus under these conditions remains uncertain.

Other factors, such as indirect interaction with wild avian species and the effectiveness of biosecurity measures, play crucial roles in influencing the dynamics of the disease occurrence. Additionally, HPAIV H5N1 RNA has been detected in environmental samples such as dust swabs, raising concerns about the potential for airborne transmission through inhalation of infectious aerosolized particles. Although H5 subtypes of AIV generally do not efficiently infect humans because they do not replicate effectively in the upper human respiratory tract and typically do not facilitate human-to-human transmission, some strains have demonstrated the ability to overcome the species barrier and infect humans, causing a range of infections from mild flu-like symptoms to severe fatal cases. Of particular interest is the capability of HPAIV H5N1 to cross the placental barrier, resulting in fetal infection. Overall, the H5 subtype is primarily a concern for individuals with direct contact with infected birds. However, the likelihood of these viruses causing large-scale human outbreaks is considered low [14]. For a long time, the scientific community researching on influenza has recognized the role of intermediate hosts, such as swine, in introducing epidemic strains into human populations. Host tropism is partly determined by the ability of HA to bind to sialic acid (SA) residues on glycoproteins on the external surface of epidermal cells infected in the respiratory and gastrointestinal tracts [44].

However, each host produces glycoproteins that are expressed on the cell surface in slightly different ways depending on the tissue. Consequently, many LPAIVs circulating in nature bind effectively only to cells of their primary host, for example, avian cells, and not to those of other animal hosts. Swine, however, seem to be capable of hosting both some AI strains and some human influenza strains, thus allowing these hosts to serve as potential “intermediates” where mixing and reassortment of viral segments can give rise to a new virus better adapted for human infection but with new antigenicity, more prone to epidemic spread and not covered by current seasonal strain vaccination. In an alternative intermediate host scenario, the ability of another mammal species to host AIVs can provide time for selection of further mammalian-specific mutations or reassortment, which predispose the virus to additional spillover and host expansion. This may explain some canine influenza infections [45]. In most spillover events among mammals, infection is presumed to occur through a predator-prey relationship, and subsequent transmission among documented hosts has either not been observed or has been very limited. This was the case in the initial infections described in seal populations in the north-eastern U.S., where numerous deaths of ducks, gulls, and related species were documented simultaneously with infection in a broad geographic area of seals. However, it remains unclear what role environmental contamination and virus persistence play in the current panzootic circulation of HPAIVs. Susceptible hosts, even in absence of a predator-prey relationship, could become infected through contamination of drinking water or through grooming of contaminated fur [13].

Wild aquatic birds serve as the natural reservoirs of AIVs and play a role in their dissemination through their long-distance migratory routes [46], infecting domestic terrestrial and aquatic birds through contaminated water or food sources [8]. However, the oro-fecal route is the primary mode of transmission among birds due to the high viral loads in the feces of infected birds, and the ability of the virus to remain transmissible for approximately 21 days [12]. One of the most commonly described risk factors for virus transmission is proximity to water, as there can be close interaction between migratory birds and commercial poultry operations, increasing the spread of the disease [47]. AIVs can also be transmitted through secretions and bodily fluids such as saliva, mucus, and urine. In production systems, these fluids and feces contaminate workers’ clothing and footwear, cages, tools, and mechanical equipment for egg collection. This transmission route is considered the main vehicle for disease spread within poultry houses, making commercial poultry farming responsible for the epidemics recorded worldwide [12].

Regarding transmission to mammals, direct contact is the main route, as effective aerosol infection of mammals by the virus has not been demonstrated. Transmission to other species typically occurs following circulation of the virus in densely populated infected avian species, indicating that AIVs can adapt and facilitate spread. For effective transmission and replication in mammals, the virus must evolve and mutate (reassortment) to achieve compatibility with the new host environment; reassortment has been responsible for the emergence of nearly all past panzootic viruses [48]. AIVs have demonstrated the ability to overcome the species barrier due to multifactorial reasons that facilitate transmission. Some mammals, such as bats [49], swine [50], cats, dogs, horses, ferrets, and sea lions can act as reservoirs, allowing for genetic mixing between viruses targeted to infect humans and birds. Furthermore, host susceptibility, exposure levels to infected birds, viral mutations, and favorable environmental conditions create an ideal scenario for zoonotic transmission of AIVs.

The main route of transmission between birds and humans is the direct contact with feces or secretions from infected animals and exposure to environments contaminated or infected with the virus. Workers involved in the poultry production chain (“from farm to table”) are at greater risk of infection. Studies showed that the virus is more resistant to low temperatures (below 28 °C). AIVs can survive up to 200 days in bodily fluids of infected birds, 4 days in feces at animal body temperature, 35 days in feces at temperatures below 4 °C, and about 5 weeks in the environment of infected poultry houses. The virus can survive in carcasses, meat, and eggs (especially at low temperatures); therefore, in cases of suspected or confirmed AI positivity, derived products must be discarded [12].

Another paradigm shift occurred in 2024 and 2025: in March 2024, H5N1 was identified in the U.S. dairy cattle, a host not previously considered relevant for IAVs [51,52,53,54,55].

Fortunately, infection in humans and cattle, unlike in other infected animal species, generally manifests as a mild disease in most cases. However, the bovine H5N1 influenza outbreak constitutes the largest known H5N1 viral infection in a domestic mammal species in close contact with humans, thereby intensifying the risk that this H5N1 variant—already adapted to mammalian hosts—may, in principle, undergo further adaptation that could increase the risk of human-to-human transmission, although such transmission has not been documented to date [56].

A diagnostic investigation was performed on samples collected by the University of Texas Medical Branch (UTMB) from affected farms, including nasal swabs, serum, and peripheral blood mononuclear cells (PBMC) from clinically affected cows, as well as milk samples. Infectious HPAIV H5N1 was successfully isolated [57]. Virus shedding was further assessed by testing milk, nasal swabs, urine, and fecal samples obtained from both clinical and non-clinical cattle. The findings indicate a pronounced tropism of HPAIV H5N1 for the mammary gland tissue, resulting in virus-induced mastitis. All HPAIV H5N1 sequences recovered from the farms in this study belonged to a novel reassortant B3.13 genotype. Following reports of mortality in wild birds (great-tailed grackles), peri-domestic birds (pigeons), wild mammals (raccoons), and domestic mammals (cats), the potential presence of HPAIV infection in these species was investigated. Whole-genome sequencing of samples from grackles, cats and raccoons from farms, confirmed infection with the HPAIV H5N1 B3.13 genotype closely related to the viruses detected in dairy cattle from the respective farms. Phylogenetic analysis indicated that the basal sequences of the viruses obtained from cats and raccoons originated from dairy cattle, consistent with cattle-to-cat and cattle-to-raccoon transmission. Epidemiological data support this interpretation, as feeding raw milk to farm cats was a common practice on these farms. The spillover of HPAIV H5N1 into dairy cattle, together with observations about the possibility of mammal-to-mammal transmission in specific ecological settings is unprecedented and raises considerable concerns, as it may drive viral adaptation and potentially enhance infectivity and transmission to humans [58]. In fact, low temperature, high humidity, and neutral-to-slightly acidic pH levels enhance virus stability, favoring the persistence in the environment and spread among susceptible hosts [44,59,60].

This “host jump” introduced new risks at the intersection of food production and public health. Dozens of human cases of conjunctivitis were reported in dairy workers, marking the first cluster of zoonotic infections linked not to poultry but to cattle [61,62]. Although the symptoms were mild, these events highlight the potential for new transmission pathways to humans and the growing complexity of AI epidemiology.

Notably, the host range has expanded over the past decade. Foxes across Europe and North America frequently tested positive, often after scavenging infected carcasses, and many have shown neurological symptoms [63]. In the U.S., infection was confirmed in two black bears and a polar bear in Alaska, broadening the host range to include ursids [60,61]. Domestic cats that came in contact with raw milk or infected preys also became ill with H5N1 positivity, sometimes exhibiting neurological symptoms [64].

In addition to carnivores, infections were documented in goats, sheep, and alpacas in South America [26,40,65,66], demonstrating that spillovers are not limited to predators or scavengers but can also involve herbivores near infected environments.

Between 2015 and 2020, most human infections were linked to direct contact with infected poultry, especially H5N6 virus in China, which caused severe illness and high fatality rates [29]. In contrast, the 2024–2025 U.S. dairy cattle-associated infections were mild but epidemiologically significant, indicating viral adaptation to mammals and emerging occupational risks [61,62]. The presence of molecular features associated with adaptation to mammalian hosts, which could facilitate further transmission following continued circulation has led many experts to consider the H5N1 virus one of the influenza viruses currently regarded as posing a substantial pandemic risk, largely due to its extensive global spread and expanding host range [31,32]. Overall, the evolution of AI epidemiology shows an increasing pattern of complexity. The virus has spread further, diversified genetically, expanded its host range, and penetrated ecosystems once considered insulated. For animal health, this has meant recurring losses in poultry industries and conservation crises for wildlife. For public health, sporadic severe zoonoses now coexist with clusters of mild occupational infections. For ecosystems, mortality events in marine mammals and incursions into Antarctica highlight the virus ability to reshape ecological dynamics [67,68].

The 2024–2025 H5N1 epizootic in the U.S. dairy cattle showed epidemiologic patterns consistent with repeated, sustained mammal-to-mammal transmission both within and among herds, including farm-to-farm spread without identifiable avian exposure and infection clusters persisting over multiple viral generations [69,70]. In contrast, the earlier introduction of H5N8 and related H5Nx viruses into mammalian hosts—such as farmed mink and wild seals between 2020 and 2022—although concerning, was characterized by more localized epizootics with limited onward spread and weaker evidence of prolonged transmission chains beyond single facilities or colonies [10,71,72]. Taken together, these patterns suggest that the cattle-adapted H5N1 genotype has crossed an epidemiologic threshold towards more efficient adaptation in mammalian populations compared to prior, spillover H5Nx events in carnivores and marine mammals.

Taken together, the available evidence suggests that the current HPAIV panzootic represents a complex and evolving One-Health challenge that requires sustained, coordinated surveillance and risk assessment [73,74].

An additional aspect of the current epidemiology of HPAIVs is the identification of molecular signatures linked to increased replication in mammals [44]. The most prominent is the PB2-E627K mutation, where Glutamic Acid (E) is replaced by Lysine (K) at residue 627 of PB2. While AIVs generally carry Glutamic Acid at this position, human IVs almost always carry Lysine, which allows for more efficient replication at the lower temperature of the mammalian upper respiratory tract [75]. The adaptive PB2-E627K mutation enables AIVs to use the mammalian infection-restriction factor ANP32A/B and trigger the NS2 enhancing activity for viral polymerase through a SUMO-interacting motif (SIM), thus acting in the adaptation of AIVs to mammals [76,77]. Another critical change is PB2-D701N, which enhances nuclear import of the viral ribonucleoprotein complex into mammalian cells and is often observed in H5N1 viruses isolated from terrestrial carnivores and marine mammals [68]. These mutations have been increasingly detected in Europe and Americas, highlighting the adaptive potential of the H5N1 virus in mammalian hosts. Their presence does not imply pandemic capacity but serves as crucial early warning signs that warrant enhanced genomic surveillance and risk assessment [74].

## 4. Geographical Distribution

Nowadays the spatial distribution of HPAIVs spans every continent. Since 2020, the H5N1 clade 2.3.4.4b virus has spread globally, and this diffusion was facilitated by wild bird migration routes. Understanding the spatial dynamics of these outbreaks is vital, as geography not only reflects viral ecology but also influences the resources and strategies for disease control [9].

Europe has become a key center of HPAIV activity since 2016, when subsequent infections by H5N8 and H5N6 led to major poultry outbreaks and unprecedented mortality among wild birds. From 2020, the introduction of the H5N1 clade 2.3.4.4b virus changed the epidemiological scenario, with year-round circulation in migratory waterfowl and recurring winter epidemics [78]. The genotype EA-2024-DI, now widespread across the continent, was identified in at least 25 countries, extending from Portugal to Bulgaria and from the U.K. to the Baltic countries and Norway [66].

Large-scale mortality events in barnacle geese, swans, and raptors highlighted the virus’s ability to impact avian species previously thought to be less susceptible [79,80]. Repeated infections into poultry in France, Hungary, Italy and Poland have caused substantial economic losses. Meanwhile, infections in resident wild birds represent a long-term challenge for eradication [81].

Asia continues to be a source of HPAIV diversity and a hotspot for the zoonotic risk. China has reported recurrent outbreaks of H5N6 and H5N8 in poultry, and H5N6 was responsible for the majority of human HPAI cases [82]. The regional intensive poultry production systems, together with live bird markets, continue to support viral persistence and reassortment [83,84]. Japan and South Korea have repeatedly recorded infections of H5N8 and H5N1 viruses through migratory birds, and extensive culling campaigns were carried out to manage outbreaks [85,86]. Southeast Asia, including Vietnam, Laos, Cambodia, Indonesia and Thailand, remains highly vulnerable, with the circulation of both H5N1 and H5N6 viruses in domestic poultry and occasional spillovers to humans [87,88].

In Africa, HPAI H5Nx viruses (i.e., viral variants or clades/genetic lineages) have spread extensively since their first detection in 2006, but the past decade has seen repeated infections associated with migratory flyways linking Eurasia to sub-Saharan regions [31]. Outbreaks of H5N1 clade 2.3.4.4b virus were reported across West Africa (Nigeria, Ghana, Côte d’Ivoire) and North Africa (Egypt, Tunisia, Morocco) [89,90,91]. Weak veterinary infrastructures and limited diagnostic capacity hampered efficient detection and control, increasing the risk the virus to become highly spread in poultry. However, regional networks coordinated by the Food and Agriculture Organization of the United Nations (FAO) and the African Union-InterAfrican Bureau for Animal Resources (AU-IBAR) have enhanced reporting and responses since 2020 [91,92].

The spread of the H5N1 clade 2.3.4.4b virus in North America in late 2021 marked a significant turning point in the geographical spread of HPAIVs. Within months, the virus was detected across the U.S. and Canada, and widespread infections were detected in wild ducks, geese, and raptors [93]. Spillover in poultry led to the culling of millions of birds, reflecting the devastating U.S. outbreak in 2015 [94,95].

The outbreaks in South American also involved mammalian species such as sea lions and alpacas, consistent with the cross-species infections previously described [65,96,97,98].

H5N1 spread in Antarctica in 2024, and the detection in brown skuas and penguins marked the virus first entry in this ecosystem [39]. The spread of HPAI into Antarctica highlights the difficult containment of a pathogen that can travel through global migratory routes [40,99,100].

**Figure 2:**
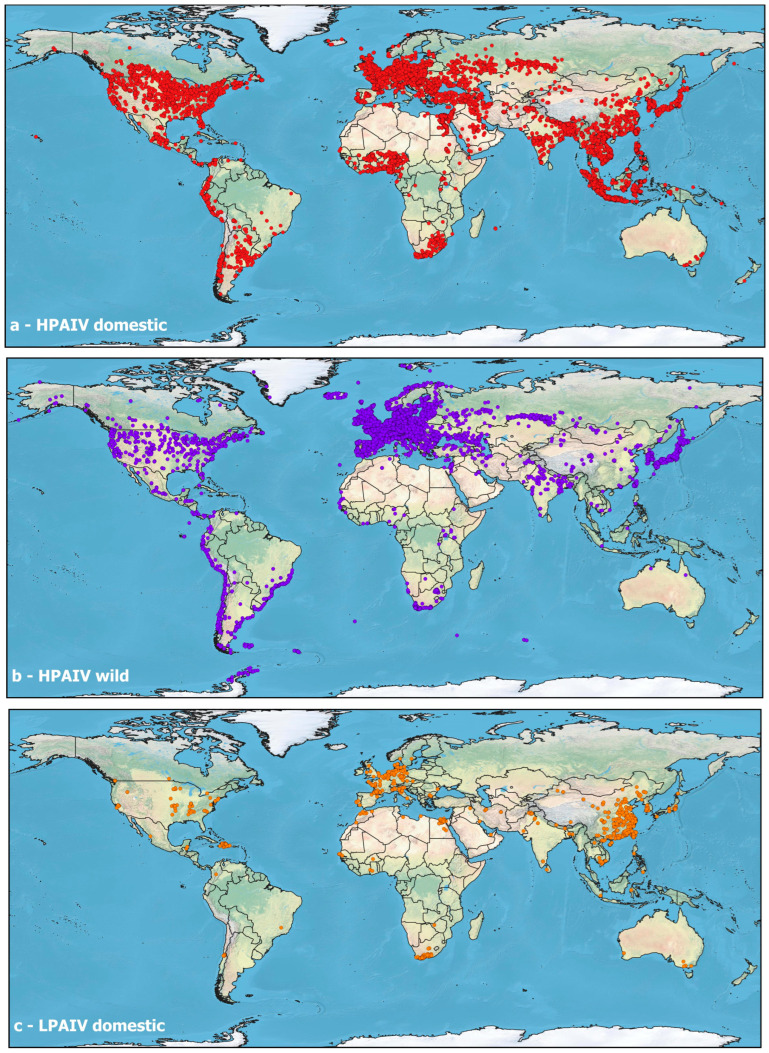

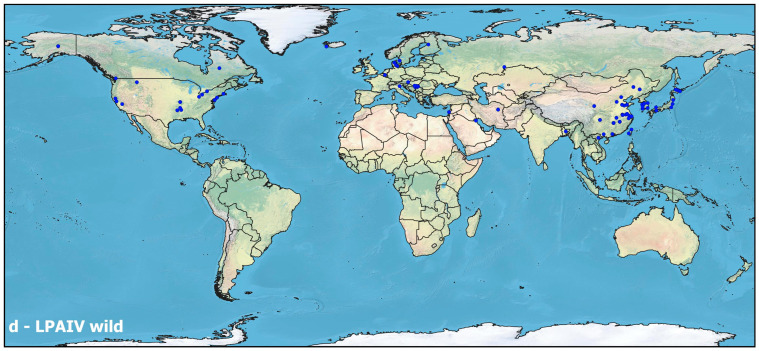
(**a**,**b**) Global geographic distribution of reported cases of HPAIV infections in host domestic animals (red spots) and wild animals (violet spots) in the time-frame 2005–2025; (**c**,**d**) Global geographic distribution of reported cases of LPAIV infections in host domestic animals (orange spots) and wild animals (blue spots) in the time-frame 2005–2025 (data source: FAO EMPRES-i+ Global Animal Disease Information System; cartography: QGIS software v.3.34.10; latest accessed on 5 January 2026).

From a geographical perspective, these events illustrate the progressive spatial expansion of HPAIVs across continents and ecosystems, rather than discrete, isolated outbreaks, and emphasize the fragility of ecosystems previously unaffected by HPAIVs. This spread has blurred the line between endemic and epidemic regions, creating a worldwide risk environment. For veterinary and public health authorities, geography is no longer a barrier but instead underlines the interconnection among ecosystems (Figure 2). Coordinated surveillance and response remain essential within the One-Health approach [54,101,102].

## 5. Pathogenesis

HPAIVs are remarkably versatile and are able to infect a wide range of cell types in many different animals. The key molecular targets for cell interaction and entry are SA receptors on the cell surface. Cell membrane-linked sialome receptors have been identified in domestic and wild birds, terrestrial-marine/aquatic-flying mammals, reptiles, amphibians and fish [11,41,103,104].

The AIV replication cycle involves several phases, starting from the binding of species-specific sialome. After receptor binding, the virus enters through endocytosis into the host cell, and the endosome originates. The fusion of host and virus membranes is possible under acidic conditions. In this phase, the viral ribonucleoprotein complex is released in the host cell cytoplasm and then the complex is transported into the nucleus. A positive mRNA template is synthetized and transported to the cytoplasm, mRNA translation is carried out in the endoplasmic rough membrane, and subsequently the virion assembly occurs in the cytoplasm. The assembled influenza viruses gather in budding zones on the apical surface of epithelial cells and exits the host cell [105,106].

The H5N1 virus triggers apoptotic cell death in epithelial cells of the upper respiratory tract of mammals [107]. In the mammary gland of dairy cows experimentally infected with H5N1, degeneration of epithelial milk-secreting cells was recorded [108].

Infection of endothelial cells by the H5N1 virus triggers excessive cytokine release and compromises the integrity of the endothelial barrier in pulmonary alveoli, leading to endothelial cell damage and resulting in a progression to viral pneumonia [109].

In birds, HPAIVs primarily replicate in the epithelial cells of the intestinal mucosa; this emphasizes the cause-effect relationship of the virus spreading through the fecal–oral route in wild birds and poultry [11,110,111]. In mammals, the virus shows a strong preference for epithelial cells lining the upper respiratory tract, enabling virus transmission through respiratory droplets and close contact [101,112,113]. These tissue-specific differences in replication correlate with typical clinical signs: digestive disease in birds and respiratory disease in mammals [11,103,111,112,113]. HPAIVs can also replicate in endothelial cells lining blood vessels in both birds and mammals. HPAIVs can also spread systemically by inducing multi-organ damages in the endothelial blood vessels [11,114,115,116].

Recently, H5N1 clade 2.3.4.4b viruses have been shown to infect mammary milk-secreting epithelial cells in dairy cows. H5N1 genotypes (e.g., B3.13 and D1.1) can replicate in the epithelial mammary gland and cause severe mammary gland inflammation. H5N1 infection of the mammary gland forms a milk route for shedding [70,117,118]. Mammary gland infection facilitates H5N1 viral transmission from the cow to the calf through raw milk nursing and potentially to other species exposed to unpasteurized milk [10,11,12,13,14]. In beef cattle, evidence suggest a typical pattern of replication in the respiratory tract: the virus is predominantly found in the respiratory parenchyma and skeletal muscle [10,11,12,13]. This divergence in tissue tropism between dairy and beef cattle underscores the virus capacity to adapt to distinct livestock [70,117,118].

The ability of HPAIVs to infect a susceptible cell type is related to the interaction between viral surface glycoproteins (especially HA) and host SA receptors. The composition and linkage pattern of these receptors vary between species and tissues within the same species [103,111,112,119]. Classical HPAIVs preferentially bind to SA with α2,3-linkages. In chickens, they bind efficiently to α2,3-linked-SA-Galβ1-4GlcNAc, whereas in ducks, they preferentially recognize α2,3-linked-SA-Galβ1-3GalNAc [119,120]. Both the bovine respiratory tract and mammary gland express numerous SA receptors; the mammary gland is rich in α2,3-linked receptors, while the bovine upper respiratory tract (especially in beef cattle) also express α2,6-linked receptors [53,119,121]. Horses show a “duck-like” pattern of α2,3 receptors in the nasal and tracheal epithelium, which supports efficient replication of avian-origin influenza viruses adapted to equids [122].

In humans, seasonal influenza viruses typically recognize α2,6-linked SA, which predominates in the upper respiratory tract, though α2,3-linked receptors are also present in the lower respiratory tract and in certain regions of the central nervous system (CNS) [94,102,103,116]. Upon adaptation to mammals, AIVs showed an interaction shift from α-2,3 to α-2,6 receptors [44].

Cats show a similar expression of α2,3-linked and α2,6-linked receptors in the respiratory tract and CNS and are susceptible to natural infection by the H5N1 virus and other HPAIVs [123,124,125]. Dogs show a predominance of α2,3-linked (“avian-like”) receptors in their respiratory tract, which is consistent with their susceptibility to several AIVs [117,120]. Pigs are classically considered as “mixing vessels” because they express both α2,3-linked and α2,6-linked receptors in many tissues of the respiratory and gastrointestinal tracts, allowing for co-infection with both avian and human influenza strains and facilitating HPAIV reassortment [103,111,126] (Table 1).

In addition to the respiratory and digestive systems, the H5N1 virus can invade the CNS in several animal species (Table 2).

Neurotropism and neurovirulence are linked in part to a multi-basic cleavage site (MBCS) in HA, which allows for efficient cleavage by ubiquitous host proteases (such as furin-like proteases) and supports systemic spread, including to the CNS [132,140]. This feature is characteristic of HP H5 and H7 viruses and is absent in LPAI strains [132,140,141]. The ability of the H5N1 virus to cross or bypass the blood–brain barrier (BBB) was proved but remains an active area of research. Studies on human H5N1 viral infections from the Hong Kong outbreak in 1997 showed that viral infection of immune cells can trigger massive production of pro-inflammatory cytokines (e.g., TNF-α) and chemokines (e.g., IP-10/CXCL10) [29,30,31]. This cytokine storm contributes to severe pulmonary and systemic damage and may also compromise the BBB integrity. IP-10 is implicated in the BBB disruption, potentially allowing the virus and/or activated immune cells to enter the CNS [104,142,143].

H5N1 viruses can also reach the CNS through neural routes. Experimental infection and pathological studies demonstrate that H5N1 virus can move through an anterograde transport mechanism through cranial nerve axons to reach the brain or other neuronal cell bodies, especially through the olfactory nerve, from the infected nasal epithelium [126,135,139,140]. H5N1 viral infection can cause neuronal death, through both necrosis and apoptosis, contributing to encephalitis and the onset of a neuro-clinical signs [144,145,146]. The respiratory tract in mammals, such as pigs, cats, dogs, and humans, expresses both α2,3-linked and α2,6-linked SAs in various CNS regions, providing potential binding sites for neurotropic H5N1 strains [113,143,147,148].

The distribution of sialome receptors in marine, semi-aquatic mammals and flying mammals (Chiroptera) explain species-specific susceptibility and their potential role as intermediate or “bridge” animal hosts. In cetaceans (whales and dolphins), there is a predominance of avian-like α2,3-linked-SA receptors in the respiratory apparatus and intestinal tract, consistent with the susceptibility to AIVs acquired through inhalation of aerosol or contact with virion-contaminated water [149,150,151]. In contrast, mustelids, such as mink, ferrets, and sea otters, show co-expression of both α2,3-linked and α2,6-linked SAs in the respiratory tract. This dual receptor sialome profile makes them potential “mixing vessels” like pigs [10,147,152]. Mustelid intestinal mucosa has predominantly α2,3-linked receptors, favoring avian-like enteric infection patterns [147,152]. Mustelids are susceptible to both avian and human-adapted HP IVs. Mustelids can act as an “ecological bridge” between wild birds and humans, creating opportunities for viral host adaptation and HPAIV reassortment (Table 3).

Flying mammals (Chiroptera) represent a special case. They host distinct influenza A-like viruses (H17N10 and H18N11) that do not use SA receptors for entry but rely on MHC-2 as receptors [160,161]. Nonetheless, many bat species do express both α2,3-linked and α2,6-linked SA in their respiratory apparatus and intestinal tract and can be experimentally infected with classical AIVs, which replicate preferentially in the gut where α2,3-linked SA receptors are numerous [160,162,163] (Table 3).

In reptiles, both avian-type (α2,3-linked SA) and human-type (α2,6-linked SA) sialome receptors were identified, and α2,3-linked SA receptors generally predominate on mucosal surfaces of the gastrointestinal tract and respiratory apparatus [46,47,48]. This double sialome receptor pattern suggests that reptiles are mainly susceptible to avian-like, intestinally related infections, making them more likely “low-efficiency hosts” or “mechanical carriers” than reservoirs for human-adapted HPAIVs. Chelonians and crocodilians show epithelial intestinal expression of α2,3-linked SA receptors, compatible with fecal–oral transmission in shared aquatic habitats [164,165,166] (Table 4).

Efficient human-to-human transmission is associated with α2,6-linked SA receptors in the upper airway; therefore the reptilian receptor sialome profile is poorly suited to sustain or amplify human-adapted influenza strains. Reptiles are “incidental hosts” that may contribute to environmental contamination with AIVs rather than “drivers” of pandemic emergence.

Amphibians and fish share aquatic environments with waterfowl, the primary reservoir of AIVs, but are not considered important hosts. Amphibians and fish possess suitable SA receptors on the skin, gills and gut epithelia. At present, there is no strong evidence for natural, productive IAV infection in amphibians and fish [48,49,50] (Table 5).

Their ectothermic physiology, innate immune defenses and other species-specific biological factors are likely to limit HPAIV replication. From an ecological standpoint, amphibians and fish are considered as “passive bystanders” or “dead-end hosts” in influenza virus-contaminated water rather than “reservoirs” or “amplifiers” of infection [186,187,188] (Table 5).

## 6. Clinical Signs

Wild aquatic birds are considered the natural reservoirs for 17 of the 19 HA subtypes and 9 of the 11 NA subtypes. However, these species are typically asymptomatic carriers of the virus [1].

Spillover events of AIVs from wild birds to domestic poultry can result in diseases ranging from mild to moderate, characterized by respiratory signs, diarrhea, and decreased egg production, to severe forms associated with high mortality. Based on the disease severity in poultry, AIVs are classified as LPAIVs and HPAIVs. HPAIV subtypes H5 and H7 are notifiable to the World Organization for Animal Health (WOAH) [189].

Eurasian H5 lineages are of particular concern to the WOAH due to their zoonotic and pandemic potential, as well as their broader host range and increased tissue tropism. These viruses are descendants of the HPAI H5N1 strain first isolated from a goose farm in China [46].

HPAIVs have become endemic in migratory aquatic birds, spreading globally and causing significant economic losses in the poultry industry [190].

Clade 2.3.4.4b viruses are responsible for severe systemic infections and high mass mortality rates in both avian species and marine mammals [1].

Although sustained transmission among mammals has not been well documented, the persistent circulation of this clade within domestic and wild mammalian populations is of great concern due to the virus adaptive potential and the risk of further spillover events. Barriers to full adaptation are mainly associated with the viral polymerase complex, which fails to ensure efficient replication within mammalian cells [1].

Due to its widespread circulation among wild birds, the H5N1 virus is considered a significant threat to the conservation of wild avifauna, including species such as cranes, Dalmatian pelicans, bald eagles, and California condors [191].

For California condors, emergency use authorization was granted for a vaccine aimed at preventing fatalities, given the high mortality rate and the critical status of the species [192].

Certain HPAIVs H5N1 clade 2.3.4.4b have been more frequently associated with infections in non-human mammals, raising serious public health concerns, as the virus has demonstrated an increased level of adaptation to mammalian hosts [193,194].

HPAI H5Nx influenza viruses have exhibited pronounced neuroinvasiveness in both avian and mammalian species—a feature rarely observed upon LPAIV infection [132].

Neurological signs are the most commonly reported clinical manifestations in mammals, although respiratory symptoms may also occur. Such neurological signs have been documented in various domestic and wild mammals, including humans [195].

Influenza viruses primarily infect cells of the respiratory tract and may subsequently spread to the CNS, likely via cranial nerves or hematogenous dissemination. In particular, HPAIVs may reach the CNS through the olfactory, trigeminal, or facial nerves. Dissemination through viremia is also possible; however, unlike in avian species, viremia and extensive viral replication are rarely observed in mammals [196]. Neurological signs are variable and may include ataxia, aggressive behavior, and visual impairment; in humans, symptoms range from malaise and headache to seizures.

Overall, clinical symptoms depend on the viral strain, the species affected, and the age of the individuals. In particular, different avian and mammalian species exhibit widely variable levels of morbidity and mortality.

### 6.1. Wild Birds (Anseriformes and Charadriiformes)

*Anseriformes* are considered among the most important reservoirs of HPAIVs. Dabbling ducks are generally more resistant than gallinaceous birds; in particular, mallards (*Anas platyrhynchos*) exhibit greater resistance, likely due to faster induction mechanisms of IFN-β production [197]. These birds show minimal clinical signs and low mortality rates. Similarly, other migratory species such as Eurasian wigeons, diving ducks, wild geese, and mute swans show limited clinical symptoms and are therefore main contributors to the spread of HPAIVs [198].

*Charadriiformes* such as gulls and terns demonstrated marked susceptibility to infection, with high mortality rates [199]. Clinical signs observed in surviving individuals include fever, depression, reduced appetite, opisthotonus, torticollis, head tremors, paralysis, conjunctivitis, dyspnea, and diarrhea [200].

In 2022, great skuas (*Stercorarius skua*) in the U.K. exhibited high mortality accompanied by neurological symptoms such as circling gait, rolling movements, and head drooping and progressing to spasticity of the head and neck [201].

Endangered African penguins (*Spheniscus demersus*) also shows high mortality and clinical signs including emaciation, torticollis, muscle spasms, incoordination, corneal opacity, and coma [202].

Pigeons and doves appear to be susceptible to infection but show a low tendency to develop clinical signs and limited capacity of viral transmission [203].

Scavenging birds and raptors may develop clinical manifestations such as torticollis, opisthotonus, neck flexion, ataxia, and circling movements, often associated with multifocal necrotizing lesions in the brain, cerebellum, and brainstem [204].

### 6.2. Poultry (Chickens, Turkeys, Domestic Ducks and Geese)

Infection with HPAIVs in chickens and turkeys is associated with high morbidity and mortality. Clinical signs arise following viral replication in various visceral organs, the nervous system, and the cardiovascular apparatus [205].

Chickens are highly susceptible to HPAIV infection and typically exhibit clinical signs indicative of pronounced neurotropism, associated with visceral and cerebral necrosis. These signs are frequently accompanied by edema of the comb, wattles, legs, and facial tissues [206].

Turkeys are even more sensitive to infection than other poultry species, showing extremely high mortality and marked neurological symptoms, including loss of balance, difficulty in feeding and drinking, tremors, and frequent diarrhea [207].

In domestic ducks and geese, intense viral replication was observed, with variable mortality rates. Clinical signs range from malaise and ruffled feathers to neurological symptoms such as abnormal head posture, tremors, and recumbency.

In ducks, the virus exhibits pronounced neurotropism, possibly due to early colonization of the CNS. Geese tend to show clinical signs associated with cyanosis of the head and hemorrhages of the conjunctiva and nares [208].

### 6.3. Mammals (Wild and Domestic)

Since 2016, HPAIVs clade 2.3.4.4b have been associated with global dissemination events involving both wild and domestic mammals and, in rare cases, humans exposed to infected birds [94]. Among wild mammals, the most affected species have been predators or scavengers such as canids and felids, as well as marine mammals. In domestic mammals, viral spread has consistently occurred following introduction by wild birds or exposure to infected poultry [209].

### 6.4. Humans

Since 2022, cases of HPAIV infection in humans have been reported. Nearly all cases were associated with exposure to infected or dead poultry, and no evidence of human-to-human transmission has been identified [210]. A single human case in Texas (USA) was linked to exposure to a herd of infected dairy cattle [211]. Clinical symptoms in humans range from mild to severe and include fever, cough, headache, fatigue, respiratory distress, diarrhea, nausea, and vomiting. Fourteen human cases have developed severe disease, and seven of them died. Genetic analyses of viral isolates revealed the acquisition of mammalian adaptation markers in those strains infecting humans and certain mammalian species. This finding raises significant concern, as such genetic changes can facilitate efficient viral replication and alter transmission dynamics both within and between species [212].

### 6.5. Pigs

Among domestic animals, pigs hold significant importance due to their role as potential “mixing vessels” for the reassortment of human and AI strains. Infected pigs are often asymptomatic; however, they are considered unlikely to serve as major carriers for the transmission of HPAIVs [209].

### 6.6. Cattle and Goats

In dairy cattle, HPAIV infection was documented, showing reduced milk production and decreased appetite, but no confirmed HPAIV-associated mortality was reported. Viral shedding was demonstrated in milk, urine, feces, and nasal swabs from infected dairy cows. In these animals, the emergence of a novel reassortant HPAIV capable of both interspecies and intraspecies transmission was observed, highlighting the virus ability to cross species barriers [55]. In goats, a slight increase in mortality rate was reported [213].

### 6.7. Farmed and Domestic Carnivores

Among farmed small carnivores, mink showed increased mortality rates accompanied by loss of appetite, depression, hypersalivation, and tremors [214].

Only a few dogs tested positive for HPAIV infection; however, cats appear to be more susceptible, exhibiting a range of clinical symptoms from subclinical forms to severe respiratory and neurological disease, sometimes resulting in death. Reported signs include anorexia, lethargy, hypersalivation, fever, dyspnea, abdominal pain, urinary incontinence, trismus, seizures, and limb rigidity [210]. Observations among animals within the same household showed no evidence of cat-to-cat transmission [215]. Additional cases were reported in cats living near infected dairy farms, most likely resulting from contact with raw milk or wild birds responsible for introducing the virus into the farm [216].

### 6.8. Marine Mammals

Marine mammals are particularly susceptible to HPAIV infection. Numerous cases were identified in seals, which exhibited clinical signs such as seizures, fever, facial spasms, and stupor [155]. Following mass mortality events among wild aquatic birds, large-scale die-offs of sea lions were documented along the Peruvian coastline. Affected sea lions showed tremors, seizures, paralysis, dyspnea, tachypnoea, nasal and oral discharge, and pulmonary edema [37].

Infections in cetaceans have been rarely reported; documented cases include a common bottlenose dolphin (*Tursiops truncatus*) and a harbor porpoise (*Phocoena phocoena*) showing evident neurological signs [156].

### 6.9. Wild Mammals

Several wild mammal species tested positive for HPAIV. A comprehensive list of detections in mammals is available through the WOAH. These animals exhibit marked neurotropism, with clinical signs including malaise and anorexia, often associated with severe necrotizing polio-encephalitis, mild myocarditis and pneumonia, as well as petechial hemorrhages in muscles, subcutaneous tissues, and visceral organs [189].

## 7. Clinical Pathology

In both wild and farmed animals, few clinical findings were reported; most observations are of pathological nature. Affected animals are generally found in good nutritional conditions, reflecting the rapid course of the disease. In migratory birds, post-mortem examination reveals congestion, petechial hemorrhages, and necrotizing lesions in visceral organs and in the brain. IHC detected viral antigen in the respiratory and digestive tracts, in the liver, pancreas, and brain. Similar lesions were reported in mammals, in which pronounced neurotropism was observed, along with petechial hemorrhages, lymphoadenomegaly, pulmonary and meningeal congestion, and necrotizing polio-encephalomyelitis [155].

In small domestic animals such as dogs and cats, some hemato-biochemical alterations and imaging findings were documented. Abnormalities include elevated alkaline phosphatase (ALP) and total protein (TP) concentration, accompanied by mild decreases in hematocrit (HCT) and erythrocyte (RBC) counts.

Thoracic radiographs may show no pulmonary parenchymal alterations; when present, imaging findings include areas of pulmonary hepatization and increased bronchial radiopacity in specific regions. Echocardiographic examinations frequently reveal mild thickening of the mitral valve leaflets with slight valvular insufficiency, while cardiac chamber dimensions remain unchanged [217].

## 8. Diagnosis

### 8.1. Clinical Diagnosis

Clinical diagnosis may strongly suggest HPAIV infection, particularly when supported by epidemiological investigations linking suspected cases to recognized HPAI outbreaks. However, clinical suspicion must always be confirmed through specific laboratory tests, as clinical presentation require differentiation from other diseases. The primary differential diagnoses in birds include Newcastle disease, avian cholera, duck viral hepatitis, septicemic colibacillosis, and other viral neuropathies. In mammals, differential diagnoses should consider rabies, distemper, pseudorabies (Aujeszky’s disease), toxoplasmosis, and other causes of encephalitis or acute respiratory compromise. For humans, relevant differentials include severe seasonal influenza, COVID-19, bacterial or fungal pneumonia, sepsis, and viral encephalitis (e.g., herpes simplex [HSV], West Nile virus [WNV]) [189,218].

### 8.2. Laboratory Diagnosis

Accurate and prompt laboratory diagnosis is pivotal for AI surveillance, outbreak control, and risk assessment. Diagnostic methods have advanced over the past decade, driven by the increasing complexity of circulating H5Nx viruses and the increased need for rapid detection at the animal–human–environment interface. While traditional methods such as RT-PCR, virus isolation, and serology remain pivotal, they are now enhanced by advanced genomic and bioinformatic tools that enable detailed analysis of viral evolution and adaptation [219,220].

Real-time RT-PCR remains the primary method for detecting IAVs. Universal assays targeting the M gene are commonly used for initial screening, with subsequent subtype-specific assays used to detect H5 and H7 [221,222]. Refinements in assay design enhanced sensitivity and reduced cross-reactivity, enabling modern protocols to detect very low numbers of RNA copies per reaction [223]. Multiplex PCR formats and portable reverse transcription-loop-mediated isothermal amplification (RT-LAMP) assays are increasingly used in field and low-resource settings, enabling faster results and reduced reliance on centralized laboratories [224].

Despite the widespread use of molecular assays, virus isolation remains the gold standard to confirm infectivity. Inoculation of embryonated chicken eggs is generally the preferred technique for virus isolation. After several days, the eggs are tested for hemagglutinating activity, and confirmation is performed by RT-PCR [58]. Virus isolation provides material for antigenic characterization, pathotyping, and the development of vaccine seed strains [225]. Although more time-consuming and requiring high-biocontainment facilities, isolation provides essential information that PCR alone cannot provide, including evaluation of viral fitness and pathogenicity. The hemagglutination assay, often used together with isolation, remains a simple yet informative tool, though it lacks specificity without molecular confirmation [226,227].

Viral load is determined by titration using serial dilutions of PCR-positive samples [63]. All samples are subjected to RT-PCR to detect the viral genome, which, through subsequent genetic sequencing, allows for identification of viral subtypes and both Eurasian and Pan-American lineages [58,228].

Serology assists in diagnosing AI, especially in epidemiology and vaccine assessment. Enzyme-linked immunosorbent assays (ELISA) are used to detect antibodies against the nucleocapsid protein [63]. Hemagglutination inhibition (HI) assays and ELISA are frequently employed to detect antibodies in poultry and wild birds, indicating previous exposure [18,229,230]. Virus-neutralization assays, although more labor-intensive, remain the most specific serological test and are increasingly employed in One-Health studies assessing the zoonotic risk [231]. In mammalian spillover hosts, serology helped detect silent or subclinical infections that would otherwise go unnoticed [232].

Various types of samples can be used, including nasal, oropharyngeal, and cloacal swabs; whole blood and serum; milk and urine; and, in deceased subjects, mammary gland, lung, lymph node, intestinal, and brain tissues. Histological specimens are typically subjected to IHC for viral antigen detection. Human and avian samples should not be processed simultaneously or, preferably, not even in the same facility, to prevent potential genetic recombination [61].

Over the past decade, the combination of high-throughput sequencing and bioinformatics has revolutionized laboratory diagnosis of AI. Next-generation sequencing (NGS) platforms enable full-genome characterization directly from clinical samples, accelerating the identification of reassortant viruses and new genotypes [233]. Metagenomic approaches have been essential in studying wildlife and mammalian hosts, where mixed infections or low viral loads can complicate diagnosis [234,235,236].

Specialized tools have been developed to facilitate standardized genomic analysis. “Genin2”, a bioinformatic tool developed by the Istituto Zooprofilattico Sperimentale delle Venezie (IZSVe), Italy, enables automated genotype assignment based on genome segment constellation criteria established by Fusaro and co-workers (2024) [31,207]. Analytical platforms such as “FluMut” can rapidly flag mammalian genetic adaptation markers (e.g., PB2-E627K and PB2-D701N mutations) for surveillance purposes [237].

Rapid diagnostic tests for AI were developed; however, these assays cannot distinguish between HPAIV and LPAIV infections. Specifically, HPAIVs possess multiple basic amino acids at the HA cleavage site compared to LPAIV strains, but current rapid tests cannot detect this difference. Novel rapid assays employing sensitive and specific nanoparticles for HPAIV detection are currently under development [238].

Emerging diagnostic technologies include clustered regularly interspaced short palindromic repeats (CRISPR)-based assays that combine isothermal amplification methods (such as RT-LAMP or RT-recombinase polymerase amplification, RT-RPA) with Cas12a enzyme-mediated detection, offering high analytical specificity in portable and field-ready formats [239,240]. Digital PCR provides increased sensitivity and more precise quantification compared to traditional real-time PCR, making it valuable for detecting low levels of influenza viral RNA and for environmental monitoring [211].

Although laboratory diagnostic capacity has increased, several challenges remain. Ongoing viral evolution requires regular updates of PCR primers and probes to maintain sensitivity. Resource limitations in parts of Africa, Asia, and South America continue to restrict access to advanced methods, emphasizing the importance of portable assays and regional reference laboratories [83]. Biosafety considerations are also essential: the isolation of HPAIVs demands specific safety measures in accordance with the WHO Laboratory Biosafety Manual, which are not universally available [241].

From a One-Health perspective, integrating laboratory data with field surveillance, wildlife monitoring, and genomic epidemiology is essential. The 2024 U.S. dairy cattle outbreak highlighted the need for rapid diagnostic adaptation to novel hosts [212]. Going forward, integrating traditional diagnostic techniques with advanced genomic technologies will be crucial for rapid outbreak identification and long-term risk assessment across diverse species.

## 9. Immune Response

### 9.1. Immunity in Birds

Wild aquatic birds constitute the natural host reservoir of AIVs and susceptible animals in which LPAIVs can replicate and spread to domestic birds such as chickens and turkeys, as well as mutate and acquire HPAIV features. Differently from mammals, birds have peculiar characteristics and diverse distribution of lymphoid organs, which are diffuse in the body, including the harderian gland, cecal tonsils, Peyer’s patches and bursa of Fabricius. Besides natural physico-chemical barriers in the body, birds react against AIVs through inflammatory mechanisms and innate soluble and cellular components. The early detection of viral infection occurs through pathogen recognition receptors (PRRs) such as Toll-like receptors (TLRs), retinoic acid-inducible gene 1 (RIG-1)-like receptors (RLRs), and nucleotide oligomerization domain (NOD)-like receptors which identify viral conserved proteins and RNA sequences [242].

The downstream activation leads to the type 1 interferon (IFN) anti-viral response and cytokine modulation. This cascade is mediated by Toll/interleukin-1 receptor (TIR)-domain-containing adapter-inducing interferon-β (TRIF) and myeloid differentiation primary response 88 (MyD88) protein which in turn activate the transcription factors interferon regulatory factor 7 (IRF7) and nuclear factor-kappa B (NF-κB). TLR3 and TLR7 are mainly involved in sensing viral single-strand RNA (ssRNA) being upregulated in several tissues such as lungs, intestine, bursa of Fabricius, spleen and, in case of HPAIVs, also in the brain. The involvement of different innate receptors can vary depending on the infecting strain (LP vs. HP strains) and determinants in the host animal. The cytosolic RLR-mediated IFN response involves a cascade through the mitochondrial antiviral-signaling (MAVS) and the chicken orthologue of stimulator of IFN genes (STING). Aldo melanoma differentiation-associated gene 5 (MDA5) and laboratory of genetics and physiology 2 (LGP2) are upregulated upon HPAIV H5N1 infection. In the respiratory tract, the NOD-like receptor pyrin domain containing 3 (NLRP3) induces inflammosome complexes that activate caspase-mediated apoptosis and pro-inflammatory cytokines (IL-1β, IL-18) [243,244].

The anti-viral activity is achieved through the expression of IFN-stimulated genes (ISGs) mediated by the JAK/STAT interaction. Type 1 IFNs bind to IFN-alpha and IFN-beta receptor subunit 1 (IFNAR1) and IFNAR2 to induce autocrine and paracrine effects. In chickens, IFN-kappa (type 1 IFN) is also involved in reactivity against LPAIV H9H2. IFN-γ (type 2 IFN) acts as strong mediator upon infection by interacting with IFN-γ receptor 1 (IFNGR1) and IFNGR2, and activating the JAK/STAT pathway. IFN-γ upregulation is associated with higher levels of type 1 IFN, Myxovirus-resistance (Mx) protein and CD8+ cells. Type 3 IFN-lambda (IFN-λ) is expressed in chickens and seems to trigger an anti-viral response in mucosae mediated by IFN-λ receptor 1 (IFNLR1) and IL-10 receptor 2 (IL10R2) [245].

ISG factor 3 for type 1 and type 3 IFNs (ISGF3) and IFN-γ activation factor (GAF) induce the expression of anti-viral genes by binding to IFN-stimulated response elements (ISREs) and gamma-activated sequence (GAS) promoter elements ISGs. Therefore, an antiviral state is established by inhibiting viral replication and modulating host anti-viral responses.

IFN-induced proteins with tetratricopeptide repeats (IFITs) are represented in chickens by a single gene (IFIT5). The anti-viral activity of IFIT5 is mediated by the ability to sequester viral RNA and inhibit transcription and translation. Upregulation of IFIT5 was observed in chicken tissues for infection with both LPAIVs and HPAIVs. Specifically, HPAIV H5N1 strains are strong inducers of the IFIT5 gene compared to LPAIVs. Chicken IFITM3 is able to restrict cell infection by IAVs and provide protection and clinical disease tolerance against HPAIV H5N1; in fact, IFITM3 inhibits HA-mediated viral entry by specifically interacting with the HA2 subunit in certain IAV strains [242].

The IFN-mediated cascade in chicken involves IFN-induced GTP-binding protein Mx as an anti-viral effector belonging to GTPases. In addition, the antiviral activity is mediated by the protein kinase R (PKR) which is an IFN-induced serine/threonine protein kinase that mediates anti-viral functions. Also viperin is an IFN-inducible protein with broad anti-viral activity, sharing motifs with the mammalian protein. Viperin is found to be upregulated in the lungs and spleen of chickens infected with HPAIV H5N1.

In chickens, the system involving the 2′-5′-oligoadenylate synthase-like (OASL) protein was found upregulated in the lungs and spleen upon HPAIV H7N9 infection. This suggests a potential anti-AIV activity of chicken OASL, like its mammalian counterpart [245].

In chickens, the pro-inflammatory response intensity and diversification is dependent on the pathogenicity of the AIV. HPAIV H5N1 is a potent inducer of pro-inflammatory cytokines in the lungs of infected chickens. LPAIVs may not induce early expression of pro-inflammatory cytokines such as IL-1β, IL-2, IL-6, or IL-8 in the lungs of infected chickens but an increase in the expression of IL-1β and IL-6 expression resulted to correspond to increased pathogenicity of the LPAIV strain. When a balanced pattern of pro- and anti-inflammatory cytokines is present, the recruitment and activation of immune cells, feedback control of cytokine production, and the acute phase response (APR) are more effective and not dysregulated like upon HPAIV infection [246].

AIV infection in chickens also induces the expression of the chemokines CCL4, CCL19, CCL10, and CX3CL1. These recruit leukocytes to the infection site and modulates cellular immunity. Although the main role of these inflammatory mediators is to activate the immune responses to control viral infection, excessive, uncontrolled, or dysregulated release can exacerbate the severity of the disease. Dysregulated cytokine levels in the lungs are associated with high fatality in mammals infected with IAV. Cytokine storm refers to the overproduction of cytokines that correlates with increased lung damage and mortality. In chickens, an excessive cytokine response is associated with severe capillary leakage in multiple organs and high mortality upon infection with HPAIV H7N1 [247,248].

Chicken endothelial cells are able to initiate a robust pro-inflammatory response to HPAIV, contributing to the cytokine storm. Moreover, the massive replication of HPAIV H5N1 in the inflammatory macrophages leads to extensive production of pro-inflammatory cytokines (IFN-β, IL-1β, IL-6 and IL-8), contributing to lung injury. The increased pathogenicity of HPAIV strains may also be associated with cytokine dysregulation resulting from virus-induced death of cytokine-producing cells such as macrophages and dendritic cells (DCs) [248].

Heterophils represent the polymorphonuclear cells in chickens and are the predominant phagocytes in the blood. They are the primary phagocytes recruited to AIV-infected tissues and express TLRs to sense various PAMPs. Heterophils also express crucial cytokines and chemokines for initiating/coordinating immune responses against pathogens; however, AIV replication in heterophils facilitates virus spread to tissues through the lymphatic system.

In chickens, CC chemokine receptors 6 and 7 (CCR6 and CCR7) are involved in trafficking of DCs to the site of infection and toward T cells, whereas glycan-binding receptors on DCs favor the attachment and entry of AIVs. Intranasal inoculation of LPAIV H7N9 results in an influx of DCs and macrophages in the lungs and upregulation of MHC-2 and CD11c. DCs are susceptible to infection by both HPAIV and LPAIV, but viral replication varies with the virus subtype. Activation and functions of DCs are also influenced by the AIV pathogenicity; in fact, DCs infected with LPAIV H9N2 increase signal transduction and innate responses but also showed impaired antigen presentation [242].

Macrophages can also produce reactive nitrogen species (RNS) such as nitric oxide (NO), which can mediate an anti-viral response. Different AIV strains can impair the NO synthesis pathway in chicken macrophages. The NS1 protein of H9N2 LPAIV inhibits Fas/Fas ligand-mediated apoptosis, thus enhancing the AIV-infectivity of macrophages. NS1 and M1 protein of LPAIV H9N2 can downregulate the expression of the immunoglobulin IgY receptor (FcRY) in macrophages, leading to immunosuppression [245].

In chickens, very low levels of NK cells were detected in the spleen and peripheral blood compared to mammals. The activation of NK cells in the lungs is associated with reduced LPAI H9N2 viral loads. The absence of NK cell activation in HPAIV H5N1-infected lungs might contribute to the increased pathogenicity of the strain.

Apoptosis of AIV-infected duck cells, compared to the relatively delayed apoptosis in chicken cells, may be involved in the resistance to AIV in ducks. However, apoptosis in epithelial cells, endothelial cells, immune cells, and skeletal myofibers is necessary for IAV replication and release. The extent of AIV-induced apoptosis in chicken macrophages varies depending on the infecting subtypes.

The bursa of Fabricius contains thousands of follicles, each having about one thousand B cells, which undergo proliferation resulting in approximately 150,000 B lymphocytes per follicle by two months of age. The bursa is essential for B cell development, supporting the diversification of immunoglobulins through somatic gene conversion. Chickens possess three subclasses of immunoglobulins: IgM, IgY, and IgA [249].

Chickens resulted to be more prone to mount a higher humoral immune response upon LPAIV H5N3 infection than ducks. When chickens are infected with LPAIV H9N2, a significant increase in their serum antibody levels begins from 5 dpi. In layers, infection with LPAIV H7N2 leads to detectable levels of antibodies in both serum and egg yolk at 7 dpi. However, antibody levels are commonly higher in serum than in yolk. Moreover, anti-influenza antibodies can remain detectable in serum and yolk of infected layers for several weeks post-infection. Chickens also produce secretory IgA on mucosal surfaces following infection with viruses that target the respiratory and/or intestinal tissues.

High levels of IgM, IgY, and IgA-expressing cells were detected in the trachea, lung, and cecum. Chickens infected with LPAIVs typically show systemic antibody levels at 7 dpi, peaking at 3–5 wpi and persisting for several weeks. Contrarily, the highly virulence and the short death time of HPAIVs can mask seroconversion in infected chickens.

The HA protein is the primary surface antigen that induces neutralizing antibodies. Mature HA, comprising HA1 and HA2 subunits, mediates receptor binding and fusion with the host cell membrane. Therefore, antibodies against HA can inhibit viral attachment to host cells. Antibodies against HA protect infected chickens from clinical disease and reduce AIV transmission, but these antibodies are subtype-specific; therefore the diversity among AIV strains within the same subtype can limit the effectiveness of neutralizing antibodies [245].

While the head domain (HA1 subunit) is highly variable across subtypes, the stem domain (HA2 subunit) is more conserved and contribute to cross-reactivity. The interaction of HA1 and HA2 subunits is essential for the presentation of epitopes on HA that trigger neutralizing antibodies and protection. NA, another key surface glycoprotein, has enzymatic activity that facilitates the release of IAV from the cell surface by cleaving SA residues. Thus, NA antibodies can counteract AIV spread by interfering with viral attachment and release from infected cells. The antibodies elicited against NA can also be protective if they match the challenge AIV subtype and are produced in high quantities.

M2 protein is a surface antigen in AIV, expressed in small amounts, and functions as an ion channel that plays a role in facilitating viral uncoating within the endosome. Therefore, antibodies against M2 could inhibit the release of the viral RNA into the cell and may offer cross-protection.

Although antibodies are also produced against inner proteins such as nucleoprotein (NP), they do not generally contribute to protective immunity.

The antibodies produced by hens due to exposure to antigens are usually transferred to their offspring through the egg. These antibodies are transferred to the embryonic circulation (mainly IgY) or swallowed by the embryo (mainly IgA and IgM) [245].

Regarding antibodies from the mother, the primary role of maternally derived antibodies (MDA) is to provide antigen-specific protection for the newly hatched chicks. Chicks hatched from breeders vaccinated with AIV inactivated vaccines exhibit high levels of HI antibodies, which confer protection against a challenge with an antigenically related HPAIV strain. Even low levels of MDA can interfere with the development of antibodies when chicks are vaccinated at an early age. However, changes in the vaccination protocol, including schedule and type of vaccines used in both parent and progeny chickens, can alter vaccine immunogenicity in young chicks with MDA [245].

The thymus is crucial for the proper development of T cells through T cell proliferation, diversification, selection, and maturation. T cells in newborn chickens are phenotypically mature but functionally immature, unable to proliferate or secrete cytokines in response to immune stimuli. In fact, full immune responsiveness develops by 1 week of age. Regarding AIV infection, the CD8+ T cell response appears to be enhanced early after infection. Upon LPAIV H9N2 infection, the percentage of CD8+ T cells in peripheral blood lymphocytes of chickens increases, together with a clear decrease in the CD4+/CD8+ T cell ratio. Low ratios also appear to persist later upon infection with LPAIVs. The early mortality associated with HPAIV infection does not usually allow for studying the kinetics of the adaptive immune responses [245].

A depleted CD8+ T cell response could also be a mechanism responsible for the increased pathogenicity of HPAIV strains. While antibody-mediated protection against AIV infection is limited by subtype/strain specificity, T cellular immune responses can provide wider protection. Cross-reactive CD8+ T cell responses to HA and NP of AIVs have been observed in chickens. Upon H7N9 infection, NK cells and KUL01+ monocytic cells increase in the lung, and KUL01+ cells upregulate MHC-2 and CD11c expression. γ/δ T cells and CD8+ T cells increase and exhibiting an activated phenotype with upregulation of CD25 expression in the lung. B cells increase in the lung and decrease in the blood and spleen, suggesting that these cells may be recruited in the periphery [250].

The AIV-specific T cell-mediated immune response can potentially be induced both systemically and locally within the major target tissues of AIV. Chickens infected with LPAIV H9N2 develop cross-reactive protective immunity against HPAIV H5N1, primarily mediated by CD8+ T cells, which declines over time. Considering the kinetics of the two T cell subsets in response to AIV infection and roles in protection, CD8+ T cells likely play a major role in the early and active control of AIV infection [245].

### 9.2. Immunity in Mice and Ferrets

Mice and ferrets are the most commonly used animal models for H5N1 viral infection. HPAIV H5N1 typically disseminates to multiple extra-pulmonary organs and infection of the CNS is associated with a lethal outcome. Immune impairment of circulating lymphocytes is an early feature of H5N1 virus lethal infection in mice and ferrets, as well as reduced numbers of CD4+ and CD8+ T cells in the lungs and mediastinal lymph nodes, associated with high levels of apoptotic leukocytes in germinal centers of the spleen [251].

Infection is generally monitored by the quantification of serum HAI titers and neutralizing antibodies; robust levels of serum IgG1 and IgG2a, and anti-HA IgG and IgA in nasal washes have been detected and can help provide a potential protection. Antigenic sites have been identified on HA of H1, H3, and H5 subtypes.

Immunocompetent mice which had a robust serum HAI antibody response 2 wpi and detectable antibodies at challenge 7 weeks later, were completely protected from lethal disease, thus highlighting the importance of B cell immune responses for protection in mice.

Both peripheral circulating and tissue B cells in the lungs are important for protection. In addition, a role was attributed to natural IgM in the early anti-viral responses and to neutralizing IgG in clearing virus infection.

Mice lacking antibodies were observed to survive to H5N1 infection, thus indicating that cell-mediated immune responses were important for protection. After challenge, protection was correlated with memory virus-specific CD8+ T cell responses [251].

Ferrets are generally accepted to be the most relevant laboratory animal model for the study of influenza virus pathogenesis and transmission [252].

Also in these mammals, HAI antibodies and neutralizing antibody titers are monitored to evaluate seroconversion upon infection. It was demonstrated that the titers in ferrets surviving H5N1 virus infection may be generally robust, depending on the dose and strain of the infecting virus.

Virus-specific IgM antibody secreting cells (ASCs) were detected within lymphocyte subsets isolated from paratracheal lymph nodes at 5 and 10 dpi while IgG ASC were higher at 10 dpi. Primary infection of ferrets with a live-attenuated H5N1 virus resulted in the induction of ASC responses in a similar time-frame to ASC responses observed in humans administered seasonal trivalent LAIVs.

In mice, the murine homolog of NKp46 (NCR1) binds HA on infected cells which stimulates activation of NK cells. NKT cells and mucosal-associated invariant T (MAIT) cells are innate T cell subsets that can significantly affect the course of IAV infections in mice.

CD4+ and CD8+ T cells reactive to seasonal human influenza viruses could cross-react with H5N1 viruses and respond by lysing target cells and secreting IFN-γ. The majority of CD4+ and CD8+ T cells were reactive to not only to NP and M1 but also to PB1, PB2 and PA. However, HA and NA were targeted by cross-reactive CD4+ T cells [251,253].

### 9.3. Immunity in Pigs

Pigs are susceptible to IAVs of human and avian origin and thus play an important role in generating new antigenically distinct reassortant IAVs when they are co-infected with human, avian, and/or swine IAVs. ANP32A, which is involved in many cell functions such as transcription, DNA repair, mRNA export, cell death, and intracellular transport, has a strong supporting role for AIV RNA polymerase compared to other mammalians’ ANP32s. However, multiple mutations and circulation of different AIVs are testified by the 2009 H1N1 pandemic IAV (H1N1 pdm09) which contains gene segments from human, porcine, and avian IAVs. At present, H1 and H3 subtypes are the predominant types circulating in humans and swine. Triple reassortant H1N1, H1N2 and H3N2 viruses typically have a common triple-reassortant internal gene (TRIG) consisting of human influenza virus PB1, AIV PA and PB2, and classical swine virus H1N1 NP, M and NS genes [254].

Pigs display clinical disease within 12–24 h after IAV exposure, which are typically mild in uncomplicated infections and very similar to humans with mild influenza disease; viral pneumonia can occur and vary in severity based on the dose and strain of the infecting virus, route of infection, age of the pig, and immune competence. Necrosis of bronchiolar epithelium, alveolar histiocytosis, and inflammatory cells in alveolar walls are primary clinical signs. Lymphocytes and macrophages early infiltrate the pulmonary interstitium and the involvement of the nasal turbinates is sometimes observed, including necrotizing rhinitis with death of epithelial cells [255].

Goblet cells are present in the upper respiratory tract and secrete mucous consisting of mucins MUC1, MUC5B and MUC5C, and various surfactants. Pigs also have large amounts of ciliated respiratory epithelial cells that remove mucous with entrapped viruses from the lungs. IAVs evading these chemical-physical barriers enter cells through SA-α-2,3 galactose and SA-α-2,6 galactose receptors distributed on epithelial cells in a similar pattern to the receptors in humans. After entering the cell, IAV particles are recognized by PRRs, including cytoplasmic RIG-1 type receptors and MDA5, as well as by endosomal TLR3 and TLR7. PRRs stimulate type 1 IFN and pro-inflammatory cytokine responses and the subsequent induction of numerous anti-viral ISGs. This leads to a reduction in virus replication and decreased viral spread. Experimental IAV infection in pigs showed an early upregulation of innate genes including cytokines and acute phase proteins (APPs). This modulation involves IFN-α, IFN-γ, TNF-α, IL-6, IL-8, IL-10, IL-12, serum amyloid A (SAA) and haptoglobin (Hp) produced by different myeloid and lymphoid cells [256].

In pig lungs, classical dendritic cells (cDC1 and cDC2), inflammatory moDCs, monocyte-derived macrophages (MDMs), and tissue resident alveolar macrophages (PAMs) can modulate and reduce viral replication. Pigs possess a subset of pulmonary intravascular macrophages (PIMs) closely attached to endothelial cells in lung capillaries where they eliminate pathogens passing through the lung blood.

PIMs may be involved in IAV-induced lung inflammation while moDCs early increase in lungs of IAV-infected pigs and secrete IL-1β and IL-8. Plasmacytoid DCs (pDCs) may help contain infection by releasing large quantity of IFN-α to inhibit virus replication and activate virus-specific CD8+ T cells. Neutrophils phagocytose IAV particles and can contribute to IAV clearance.

The reduction in NK cells in the blood was associated with a concomitant increase in the lung where NKp46+ activated NK cells increase in areas of IAV-infected cells.

T cell subsets including γ/δ T lymphocytes, invariant natural killer T (NKT) cells, and mucosa-associated invariant T (MAIT) cells are involved in the immune response to infection. CD3+NKp46+ NKT cells were seen to proliferate in pig lungs, suggesting that these cells are involved in controlling IAV during early infection. CD2-γ/δ+ T cells are recruited to the site of infection by chemotaxis induced by CCR5 where they produce pro-inflammatory cytokines, especially IL-17. They also kill infected cells through cytotoxic/non-cytotoxic mechanisms and recruit NK cells and neutrophils. Also T cells producing perforin and IFN-γ are early recruited in the nasal mucosa and lung tissue [254].

IAV clearance is associated with the emergence of both IAV-specific T and B cells. IAV-reactive effector T cells are detectable in the blood, lung lamina propria, lung airways, and tracheal-bronchial lymph nodes. The highest frequency of polyfunctional memory CD8+ IAV-specific T cells is detected in the lung. Mucosal anti-viral IgA can be measured in the nasal cavity after inoculation and in oral fluids. Peripheral anti-IAV IgG are induced early after infection, and by 1 week, anti-NP antibodies are detected in serum together with HI antibodies. Although IAV-specific IgG and IgA antibodies exhibit reactivity to homologous strains, they usually do not possess wide cross-reactivity. Complement-mediated cell cytotoxicity, antibody dependent cellular cytotoxicity (ADCC), and antibody-dependent cell phagocytosis were observed and related to clearance and protection [254].

### 9.4. Immunity in Cattle

Experimental infection studies were performed in calves and lactating cows by using HPAIV H5N1 clade 2.3.4.4b administered oro-nasally and directly into the mammary gland, respectively [127].

Inoculation of calves resulted in moderate nasal replication and shedding with no severe clinical signs or transmission to sentinel calves. In dairy cows, infection resulted in no nasal shedding but severe acute infection of the mammary gland, with necrotizing mastitis and high fever. Milk production was rapidly and markedly reduced, and the physical condition of the cows was severely compromised. Therefore, milk and milking procedures, rather than respiratory spread, are likely to be the primary routes of H5N1 bovine-to-bovine transmission [56,128]. Specific antibodies were detected from 7 dpi onwards. Also heifers resulted susceptible to infection and showed seroconversion upon inoculation by an aerosol respiratory route [257].

### 9.5. Immunity in Horses

In horses, the main equine-infecting H3N8 and H7N7 viruses are believed that they originated and evolved from birds. The evolution of the H3N8 subtype was driven by antigenic drift, that is the inherent ability of HA and NA genes to mutate and accumulate point mutations over time [258]. Upon infection, EIV replicates in the epithelial cells of the nasopharynx and trachea inducing inflammation, which leads to erosion of the ciliated epithelium and nasal discharge. Usually, EIV infection is responsible for acute cytopathic infection but induces long-lasting immunity that is not strictly linked to the presence and levels of circulating antibodies to viral HA [259]. Even horses with low or undetectable circulating antibodies were found to be protected from re-infection. Immunity in these horses is characterized by absent or markedly reduced clinical signs and virus excretion. EIV infection induces large amounts of virus-specific secretory IgA in the nasal secretions that neutralize infectious viral particles as well as cellular immune responses, resulting in the induction of cytotoxic T lymphocytes (CTLs) able to kill infected cells. Virus infection can induce long-term CTL immunological memory; upon re-exposure to EIV infection, the rapid expansion of memory CTLs acts together with anamnestic antibody responses to destroy virus-infected cells and infectious free virus, respectively [260,261].

### 9.6. Immunity in Non-Human Primates

Experimental infection in non-human primates with a bovine-adapted HPAIV H5N1 clade 2.3.4.4b and the human-adapted (PB2-E627K mutation) huTX37 H5N1 strain (B3.13 genotype, clade 2.3.4.4b), isolated in Texas (USA) in 2024, was studied in cynomolgus and rhesus macaques by intranasal and intratracheal (IN + IT) routes [262,263]. The resulting high viral replication and severe respiratory disease was associated with a wide and acute dysregulation of the pro-inflammatory and innate immune responses. Immune cell exhaustion (i.e., immune cells becoming dysfunctional and unable to properly fight infection due to persistent activation) and apoptosis together with the reduction in NK cells, T cells and B cells was observed. However, the relatively low humoral antibody response could sustain a certain degree of protection upon re-challenge [263].

### 9.7. Immunity in Humans

Strong and multi-factorial inter-specific barriers usually prevent human infection by AIVs. However, the occasional zoonotic transmission (H5N1, H9N2, and H7 subtypes) of AIV infection to humans can happen and is usually self-limited and variable in severity. Investigation in healthy volunteers experimentally infected by the intranasal (IN) route with AIVs (e.g., H1N1, H3N8, and H3N2) showed the onset of mild clinical signs and variable antibody responses, demonstrating that humans can be infected by AIVs but the infection efficiency is low [14,264,265,266].

On the contrary, AIV H5N1 replication in animals could lead to additional mutations (reassortment or antigenic shift) that may increase human infectiousness (human adaptation) and its pandemic potential [267]. Variable disease occurrence and outcomes were observed in naturally infected humans, from influenza symptoms (respiratory and intestinal) to severe pneumonia and fatal cases (acute respiratory distress syndrome [ARDS] and multi-organ failure). These variable clinical signs depend on the features of the virus that in some cases can be directly transmitted to humans from poultry, becoming highly pathogenic (i.e., augmented virulence and low survival of the human host) [268].

Primary human cell cultures have been used to study the innate immune responses against IAV, and H5N1 in particular. Specifically, primary human tracheo-bronchial epithelial (HTBE) cells, primary bronchial epithelial cells (BECs) in an air-liquid interphase and pseudo-stratified and polarized cultures containing ciliated, secretory, and basal cells resembling human airway epithelium were utilized. It was demonstrated that AIVs and human influenza viruses infect different cell types as a consequence of their different receptor specificity (α2,3-SA and α2,6-SA) in HTBE cells, as well as that type 1 and type 2 alveolar pneumocytes respond to human and avian IAV by cytokine release. The type 1 IFN response in H5N1-infected cells is however delayed and upregulation of several cytokines/chemokines (e.g., IFN-α/β, IFN-λ1, TNF-α, IL-1β, IL-6, IL-7, IL-8, IL-12, RANTES, MCP-1, MIP-1, TRAIL, CCL2, IP-10) and mediators (e.g., TLR3, RIG1, MDA5, ISGs) was detected in several cell models [269].

Primary human lung micro-vascular endothelial cells (HMVECs) support viral replication of H5N1 viruses and infection results in strong up-regulation of pro-inflammatory genes. Human umbilical vein endothelial cells (HUVECs) showed that H5N1 viruses induced strong inflammatory responses, mostly mediated by activation of NF-κB, supporting a possible role for endothelial cells in the cytokine storm in infected individuals.

In addition, IAVs, and specifically the H5N1 strain, infect human monocytes, macrophages, and human MDMs by inducing a high production of cytokines. Such high levels of pro-inflammatory mediators were also observed in epithelial cells, which may be involved in the hyper-induction of cytokines in the lung observed upon H5N1 IAV infection [270]. While human mDCs are also susceptible to infection, pDCs seem to be resistant to H5N1 viral infection.

It was proven that the H5N1 strain is able to directly infect human NK cells by interacting with the NKp44 and NKp46 receptors and activate them to express CD69 and kill infected cells. Macrophages and DCs, due to their expression of PRRs, can contribute to the induction of the severe damage in infected humans by recruiting monocytes and neutrophils and stimulating the cytokine response in epithelial and endothelial cells. These cytokines have severe damaging effects on airway and lung tissues. The oxidative stress mediated by ROS and other cytotoxic mediators as a consequence of an acute innate immune response is further involved.

The interaction of HA with cell receptors can contribute to the induction of cytokine production and the activation through JAK2/JAK3 and STAT/NF-κB in activated B cells, which correlates with the induction of IP-10, IL-6, IL-8, MCP-1, MIP-1α, MIP-1β and RANTES. Virus infection was reported to activate tyrosine kinase (TK) receptor, p38 mitogen-activated protein kinase (MAPK) and extracellular signal-regulated kinase (ERK) [269].

The H5N1 strain NS1 protein plays multiple functions during cell infection, mostly related to its inhibition on type 1 IFN responses and NF-κB activation. It prevents activation of the 2′-5′ OAS/RNase-L pathway and of the protein kinase RNA-activated (PKR). Also, the dsRNA binding domain mediates the interaction with RIG-1, TRIM-25 and PKR, resulting in an altered anti-viral response mediated by ISGs and increased viral replication. Additionally, IAV NS1 was reported to alter the host translational machinery to enhance viral translation and can result in subsequent Akt activation through PI3K. NS1 is an innate immune inhibitor and an important virulence factor for IAV also because it induces apoptosis and necroptosis in human monocytes [271].

H5N1 viral polymerase PB1-F2 interacts with MAVS, reducing the mitochondrial membrane potential and inhibiting the RIG-1-mediated IFN-α/β secretion. PB2 was also reported to induce TNF-α, IFN-β and IP-10 in human primary macrophages and type 1 pneumocytes infected with HPAIV H5N1 and therefore is thought to contribute to the increase in cytokines in serum in H5N1-infected patients [269].

Efficacious T cell adaptive immunity relies on efficient and diversified MHC-1-mediated presentation of viral determinants to CD8+ T lymphocytes while AIV replicates in the cytoplasm. Activated CD8+ CTLs can destroy virus-infected cells by perforin-granzyme action or pro-apoptotic pathways. Simultaneously, APCs including DCs, macrophages, and B cells can interact and process released virions. The processed viral peptides presented on MHC-2 are recognized by CD4+ T-helper cells. When B cells are activated by cytokine release from CD4+ T lymphocytes, they secrete antibodies against the virus. The generation of strong and antigen-specific B and T cell immunity is fundamental for developing effector CD8+ T cells and a CD4+ and CD8+ T cell memory. Variable levels of serum HAI and neutralizing antibodies were detected upon H5N1 infection in humans during the convalescent stage [251]. HPAIV H7N7 and H7N9 infections were demonstrated to trigger different strain-specific and cross-reactive antibodies detectable in serum and memory B cells, and high antibody responses persisting for long periods, respectively [244,272]. T cell cross-recognition against H5N1 viruses was seen to be related to CD8+ T cell epitope conservation and this may provide some protection in humans [273]. During H7H9 infection, highly activated circulating CD38 + HLA-DR + PD1 + CD8+ T cells were detected and associated with protection [274].

H5N1 infection in humans was seen to be associated with hyper-induction of cytokines, defined as hypercytokinemia or cytokine storm, as well as immune dysregulation, including leukopenia (lymphopenia and thrombocytopenia) in the spleen and lymph nodes, and tissue damage. In the lungs, apoptosis of alveolar epithelial cells and leucocyte infiltration were detected [251,264,275]. The severity of the disease is also attributed to the ability of the HP viruses to evade the anti-viral immune response and thus to the ability to replicate and spread in many tissues of the human host.

## 10. Development of Vaccines

The development of efficacious vaccines in poultry and humans is related to the capacity of LPAIVs to acquire characteristics of HPAIVs due to subsequent mutations during infection and adaptation to new hosts. The emergence of new variants and strain resistance necessitates the change in vaccine seed strains to antigenically match the circulating field strains over time [241].

Therefore, mass vaccination in poultry is still an effective approach to confer immune protection and limit the spread of AIV strains as well as to reduce severe illness. Some AIV vaccines have been licensed for use in poultry and others are under development to become more efficacious, also in case of inter-specific spillover events. IVs having reservoirs in animal hosts have been circulating and had the capacity to infect humans on several occasions [276,277]. It is believed that strong stimulation of the immune system in poultry may increase the viral mutation rate due to immune pressure. Infected animals develop antibodies against HA and NA, which are highly immunogenic but at the same time prone to antigenic drift which, due to gradual changes in amino acid composition of the epitopes, leads to altered recognition and eventually to immune escape [278].

Inactivated AIV vaccines have been extensively used but the continuously evolving viruses encompassing numerous subtypes, clades and sub-clades, force to develop antigenically renewed vaccines [279]. This kind of vaccines is produced in chicken embryonated eggs but sometimes the resulting vaccines can have reduced immunogenicity due to the technological process. Therefore, cell cultures (Madin-Darby canine kidney [MDCK] and African green monkey kidney [Vero] cells) have been employed for virus amplification and to avoid epitope mutation, thus improving immune protection and efficacy in clinical trials. Usually the purified virions are inactivated and then emulsified with oil-based adjuvants; this was achieved for an H5N1 vaccine to induce higher levels of cytotoxic CD8+ T cells and cytokines. Vaccine inactivation has also been used when vaccines were produced using reverse genetics which enables the assembling of viruses containing internal proteins of non-pathogenic AIVs and displaying surface determinants of HPAIVs [280]. To trigger seroconversion and the development of immune responses, live-attenuated vaccines for humans are available in the U.S., Canada and Europe. Vaccines derived from cold-adapted and temperature sensitive donor viruses are amplified in eggs, causing egg-adaptive mutations in HA, so that tissue-restriction can occur, and they mimic natural infection. IgA and IgG are induced and detected in the respiratory mucosae, blood and other biological fluids. Chimeric vaccines have used the backbones of other viruses (e.g., fowlpox, Newcastle disease virus [NDV], and turkey herpesvirus [HTV]) to express HA and NA genes for protection against H5N1 and H7N1 viruses. By reverse genetics, an NDV-based vaccine, expressing H5N1 virus HA genes, induced a strong HI antibody response in chickens [275,280].

Virus-like particles (VLPs) are highly safe systems as vaccines, as they are non-infectious particles produced in bacterial, yeast, insect or animal cells. They show antigenic features resembling those of the original pathogen; therefore they can be used as delivery systems or antigens. They can stimulate innate immunity in terms of DCs as well as trigger adaptive B and T cells. VLPs have been used for the production of vaccines for H5N1, N3N2, H9N2 strains composed of HA, NA, and M1 proteins. The immune responses detected included HI and neutralizing antibodies associated with reduced viral titers and replication and reduced clinical signs in chickens, mice and ferrets [276].

DNA vaccines are based on the genetic code for antigens inserted in a non-replicative eukaryotic expression vector, often adjuvanted by a liposomal system, which can be delivered into the host cell by gene transfer. These types of vaccines usually induce Th1 cellular responses, more that humoral responses, thus mimicking the viral interaction with the host tissues. The antigen is processed inside the cell and peptides are presented by MHC-1 or MHC-2 molecules to stimulate CD8+ CTL and CD4+ cell responses, and activate B cells to evolve into plasma cells producing antigen-specific antibodies. Larger animal models, such as ferrets and cynomolgus macaques, offer more pertinent data as they are susceptible to human influenza viruses. Especially ferrets showed clinical signs, lung pathology, and transmission similar to humans, while cynomolgus macaques showed human-like immune responses to influenza, making them efficient predictors of vaccine efficacy in humans [276].

mRNA-based vaccines, also used during the COVID-19 pandemic due to severe acute respiratory syndrome coronavirus 2 (SARS-CoV-2), in which mRNA for HA, NP, and NA is encapsulated in lipid or chitosan nanoparticles (LNPs), demonstrated to be efficacious in stimulating CTL immunity upon infection with IVs in mice, ferrets, pigs and chickens. While conventional non-replicating mRNA vaccines were shown to prevalently induce neutralizing antibodies, self-amplifying mRNA (sa-mRNA) enhance T cellular responses and provide higher protection [276,281].

Also DC-targeted vaccines have been studied in order to deliver influenza antigens to DC receptors to directly initiate a cellular cascade that stimulates antigen-specific lymphocytes in lymphoid tissues or in lymph nodes and induce a more prolonged immune response [276].

For antigen targeting and optimizing vaccine design, deep learning approaches were explored to predict antigenic drift in H5N1 HA variants, to find viral escape mutations and epitope binding affinity and immunogenicity. Furthermore, neural network-based models were used to optimize mRNA vaccine sequences for enhanced expression and immunogenicity [267].

Swine IAV vaccines are mainly adjuvanted, whole-inactivated virus (WIV) vaccines administered intramuscularly. They provide limited efficacy against infection/transmission, and protection is often restricted to homologous strains, or antigenically similar viruses, while efficacy against heterologous strains is usually low. Immunogenicity and protective efficacy are often related to the ability of inducing virus-specific CD8+ tissue resident memory T cells, but immunogenicity can be negatively affected by MDA levels at vaccination in piglets. Live-attenuated influenza virus (LAIV) vaccines can be highly efficacious and can provide a degree of hetero-variant and hetero-subtypic protection, even when delivered in the presence of MDA. However, they can reassort with circulating endemic viruses to generate new variants. Mucosal-delivered vectored vaccines combined with efficacious adjuvants can induce protective immunity. Also in pigs, like in humans, neutralizing and HI antibodies, together with secretory IgA, are used as indicators of protection and cross-protection [254].

In the horse, the ability to induce an EIV-specific antibody response is a correlate of protection that helps limit the spread of the disease and viral shedding during an outbreak. The surface glycoproteins (HA and NA) of EIV are immunogenic and play pivotal roles in mediating infection and viral spread. EIV-specific neutralizing antibodies directed against HA or NA represent the first adaptive defensive response against virus infection or immunization. The induction of EIV-specific neutralizing antibodies supports the neutralization of the virus attachment to respiratory epithelial cells (anti-HA antibodies) and block the virus spread from infected cells (anti-NA antibodies). Cell-mediated immunity, which involves cytokine-mediated induction of antigen-specific CTLs, NK cells, and macrophages, is of major importance for protection against infection. Over the years, different vaccine technologies have been employed for equine vaccination against EIV: whole inactivated virus vaccines, subunit vaccines, modified-live attenuated, DNA-based and recombinant vector-dependent vaccines have been developed and used for intramuscular and intranasal administration [259,261].

## 11. Pathology

Gross pathology of HPAIV H5Nx in both avian and mammalian species is closely related to its cellular tropism, which varies according to host species and target cell type. In some cases, the tropism is relatively symmetric (neurotropism) where the virus preferentially infects neuronal cells, resulting in neurological signs and characteristic neuropathological lesions across different animal species. In other cases, the tropism is asymmetrically evident: for example, endothelial cells are major targets in birds, leading to widespread hemorrhages and edema, whereas the endothelial cells in mammals are less involved; therefore the hemorrhagic lesions are petechiae. HPAIV H5Nx display species-specific tropism, as demonstrated in dairy cattle, where the virus infects milk-secreting epithelial cells in the mammary gland. This recent H5N1 viral tropism suggests a potential route of viral excretion through the milk. The interplay between the viral tropism and host-specific factors contributes substantially to the observed spectrum of gross pathology, ranging from multi-organ multifocal hemorrhages, necrosis, and edema in birds to organ-specific lesions. In mammals, primarily the respiratory system and the CNS (including mammary glands in dairy cattle) are the most relevant anatomic sites of pathology [198,282,283].

LPAIV strains were also shown to replicate in reptiles. Experimental studies using embryonic tissues from American alligators (*Alligator mississippiensis*) demonstrated that LPAIVs can replicate in embryonic fibroblast cultures and in embryonated eggs. These findings indicate that reptilian cells can support viral entry, transcription, and replication, although the extent of viral shedding remains uncertain. Alligators may act as potential intermediate hosts or reservoirs for LPAIVs under certain ecological conditions, underscoring the importance of understanding cross-species viral dynamics beyond traditional avian hosts (e.g., from reptiles to amphibians) [170].

### 11.1. Avifauna

**Figure 3:**
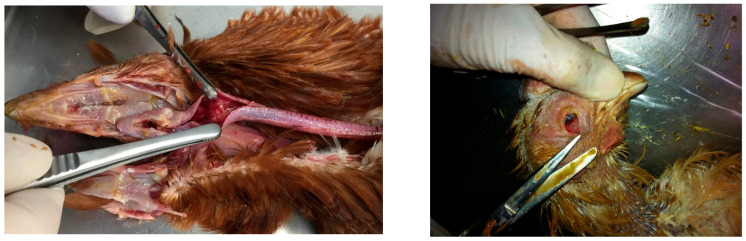
Avian influenza virus H5N1 naturally infected chickens showing acute diffuse hemorrhagic tracheitis (**left**) and acute hemorrhagic conjunctivitis (pink eye) (**right**). Courtesy of Dr. G. Tosi—Istituto Zooprofilattico della Lombardia e dell’Emilia-Romagna (IZSLER)—Forlì Regional Office, Forlì, Italy.

HPAIV H5N1-9 affect domestic poultry, wild birds, and seabird populations worldwide [234]. In domestic flocks, HPAI outbreaks have epidemiological features of high morbidity and high mortality. H5N1-infected avian domestic species show respiratory distress, neurological signs, edema and multi-organ hemorrhagic lesions (Figure 3).

Wild birds, especially waterfowl, serve as natural reservoirs and vectors for AIVs and allow HPAIVs to spread over long distances, along migratory routes and in many latitudes [96,234,284,285]. In wild birds, raptors show a strong susceptibility to HPAIV infection, often displaying pronounced neuro-clinical signs and brain lesions. In raptors, CNS lesions are frequently severe and diffuse, affecting different brain anatomical regions. The high severity of CNS pathology in raptors underscores their susceptibility compared to other avian species and highlights the neurotropic nature of certain HPAI strains in raptors. Seabird colonies are particularly susceptible due to high-density nesting, which can facilitate HPAIV transmission inside the rookery with high morbidity and high mortality trends [96,284,286].

Natural infection with HPAIV H5N1 clade 2.3.4.4b was detected in *Humboldt penguin* (*Spheniscus humboldti*) tissues collected post-mortem from asymptomatic individuals in a British zoological setting. Upon external examination, the carcasses showed systemic congestion of mucous membranes. Lungs were affected by diffuse interstitial pneumonia associated with pulmonary edema and congestion. Mild hepatomegaly, with gallbladder distention, and pancreatic petechiae were also observed. Histopathology revealed acute multiorgan inflammation with parenchymal necrosis in the lung, liver, and spleen. H5N1 viral antigen was detected by IHC in endothelial cells of the blood-vasculature of the lung, spleen, liver, brain and heart. Lymphocytes also tested immunopositive to H5N1. Immunolocalization of H5N1 antigen was performed in the lungs, spleen, liver, brain endothelial cells and in the cardiac muscle, as well as in lymphocytes, providing strong biological evidence that endothelial cells and lymphoid cells are pivotal cells for viral replication [287].

The severity and distribution of lesions depend on the clinical progression of the HPAIV infection and the avian species affected. Wild birds often exhibit mild or localized lesions, whereas chickens and turkeys typically develop a severe clinical course characterized also by a diffuse systemic vascular damage. H5N1 clades have been associated with unusual high mortality in wild aquatic birds, highlighting a shift toward increased virulence and severity of lesions within a broader host range for locally, or globally, circulating viral lineages or clades [288].

HPAI peracute cases are characterized by sudden and high mortality within affected avian populations. Birds often die asymptomatic in gross external body congestion. Birds show marked cyanosis of the comb and wattles, accompanied by edema of the head and neck [198,289,290,291].

Gross pathological changes are characterized by diffuse multi-organ vasculopathies in which congestion and hemorrhages affect the splanchnic serosal surfaces, coelomic fat, epicardium, and the mucosa of the proventriculus. Vascular lesions are also recorded as hemorrhages of the pericardium and multifocal myocardial petechiae. In domestic avian species, particularly chickens and turkeys, the HPAIV infection evolves into a severe systemic vasculopathy [198,292].

Histopathology reveals diffuse blood-vascular damages, characterized by endothelial cell necrosis, thrombi formation and multifocal-to-diffuse hemorrhages, often resulting in secondary ischemic multi-organ disorders. Blood endothelial damages are pivotal to the pathogenesis of HPAIV infection. IHC and in situ hybridization (ISH) recognize viral antigen in the cytoplasm of endothelial cells, supporting the fundamental role of HPAIV vascular tropism in disease onset and progression [293].

HPAIV infection causes lesions in the lungs and air sac. The lung parenchyma appears cyanotic and edematous, while inflammatory conditions in the air sac alveoli (airsacculitis) are observed. Multiple organ hemorrhages, including those with fibrinous exudate, result from severe endothelial damage caused by viral replication (lytic cycle) in endothelial cells. In ducks and some waterfowl, lesions are found primarily in the gastrointestinal and respiratory tracts [100].

Histopathological changes in the upper airways, observed in increasing order of severity, include: (1) desquamation of the bronchial epithelium associated with heterophilic infiltration; (2) bronchitis/bronchiolitis; (3) necrotizing tracheitis. As the infection progresses and extends deeper into the respiratory tract, into the respiratory gas exchange system (alveoli), a pulmonary response of interstitial pneumonia occurs [100].

HPAI H5N1-9 viruses are responsible for multi-organ necrosis due to the lytic cycle in target cells where viral replication takes place. Multifocal-to-coalescent foci of necrosis and hemorrhages in pancreas, liver, spleen, renal parenchyma and occasionally in myocardium are recognized [293].

Histopathology of lymphoid organs, including the bursa of Fabricius, thymus, and spleen, reveals marked lymphoid necrosis accompanied by marked lymphoid tissue depletion. These changes reflect the strong lymphotropism and immunosuppressive effect of HPAIVs. In the bursa and thymus, and in the cortical and medullary regions, the HPAIV histolesivity effect results in a diffuse lymphocytic apoptosis and lymphocyte necrosis, leading to disruption and atrophy of lymphoid organs. In the spleen, multifocal areas of lymphocyte necrosis and depletion of periarteriolar lymphoid sheaths are observed, frequently associated with blood sinus congestion and hemorrhages of the red pulp. The extensive damage of lymphoid organs compromises the host immune responses, facilitating viral diffusion inside the body and contributing to the high mortality observed in susceptible avian species [198,294].

In ostriches, gross pathological findings are predominantly associated with inflammatory processes affecting the peritoneum and liver. The peritoneum often shows fibrinous-to-fibrino-hemorrhagic fluid collection, sometimes accompanied by visceral serosal—parietal serosal fibrous adhesions to adjacent organs. The liver commonly appears megalic, due to blood stasis, often associated with multifocal necrotic or hemorrhagic areas, reflecting both direct viral replication damage and secondary hemodynamic disorders (congestion). These lesions are less extensive in the liver parenchyma of gallinaceous poultry [295].

The neurotropism of HPAI H5N1-9 viruses results in the onset of neurological signs and corresponding brain lesions. Wild birds are generally more prone to neuroinvasion than domestic avian species, reflecting differences in host susceptibility. Clinically, affected birds show muscular tremors, ataxia and other motor disturbances, as well as encephalitis. Multifocal parenchymal hemorrhages, neuronal necrosis and gliosis, often accompanied by perivascular cuffing and inflammatory cell infiltration are recorded. Raptors show a heightened susceptibility; frequently CNS lesions are severe and diffuse. These findings highlight the critical role of neurotropism in the disease outcome and the ecological impact of HPAI outbreaks on predatory avian populations [289].

### 11.2. Mammals

Terrestrial, aquatic and flying mammals, whether domestic either free-ranging or in zoological setting, show variable susceptibility to HPAIV H5N1 infection [96,296].

Within this context, members of the order Carnivora, specifically the suborders Feliniformia and Caniniformia, are reported as highly vulnerable to HPAIV H5N1 infection [297,298].

This susceptibility was documented both in free-ranging populations, where natural exposure occurs, and in zoological settings, where outbreaks often lead to severe morbidity and mortality [299,300]. In contrast, domestic ruminants (dairy cow, beef, sheep, goat) and camelids (alpaca) and other livestock (pig) may also be affected, but their body and immune response to HPAIV H5N1 appears more heterogeneous, with interspecies differences influencing both infection dynamics and clinical or asymptomatic outcomes [301,302].

Such variability highlights the importance of species-specific factors in shaping the epidemiology of HPAIV H5N1 across mammalian receptive hosts [303].

#### 11.2.1. Humans and Non-Human Primates

In humans, H5N1 viral infection induces severe immune homeostatic disorders, secondary to cytokine activation, induces lymphoid depletion in lymphoid organs, hemophagocytosis and myofibrilllary necrosis in striated muscles. H5N1 virus replicates in alveolar epithelial and endothelial cells. Viral replication in the endothelial cells induces cellular lysis with diffuse viral body invasion [304].

Gross pathology is related to acute necrotizing interstitial pneumonia histopathologically characterized by interstitial pneumonia, bronchiolitis and respiratory syncytial cell formation [305].

Cynomolgus macaques (*Macaca fascicularis*) is susceptible to the H5N1 virus, strain A/Hong Kong/156/97 and huTX37-H5N1 or H5N1 clade 2.3.4.4b. Infected non-human primates are affected by acute necrotizing interstitial pneumonia, alveolar septa thickening, alveolar edema, fibrin alveolar deposition, moderate to marked infiltration of macrophages and neutrophil granulocytes. H5N1 antigens are immunolocalized in alveolar macrophages, type 1 and type 2 pneumocytes, bronchiolar and bronchial epithelial cells and neutrophil granulocytes. Immunopositive lymphocytes are also found in the palatine tonsils [148,262,263]. The oro-gastric H5N1 infection route is not able to induce a clinical course but seroconversion is detected [262].

#### 11.2.2. Carnivora—Domestic and Wild Felines (Cats)

Coleman and co-workers in 2025 reported that “607 AIV infections in felines, including 302 associated deaths, comprising 18 countries and 12 felid species” had been recorded from 2004 to 2024 [64].

HPAIVs associated with feline mortality were recognized as subtypes H5N1, H5N6, H7N2 and H9N2. H5N1 shows case CFRs of 71% in wild felines and 52.8% in domestic cats, whereas H5N6 and H9N2 infection is uniformly lethal (100%) in both feline groups and 50% lethal for H7N2. No cases of death were recorded in Feliniformia infected with H3N8. H5N1 clade 2.3.4.4b viral infections account for 90% of deaths in wild cats and 89.6% in domestic cats. An epidemiological worldwide investigation pointed out that H5N1 clade 2.3.4.4b was detected in domestic cats and wild felines in many European Countries (France, Poland, Italy), in North America (U.S.), in South America (Peru) and in Asia (South Korea) [64,70,210,215,217,306,307,308].

At necropsy, a moderate-to-severe pulmonary congestion and edema are observed together with interstitial pneumonia, whereas no gross lesions are detected in the CNS or gastrointestinal tract. Histopathology reveals prominent interstitial pneumonia and alveolar infiltration by macrophages and neutrophil granulocytes. Neuropathology shows multifocal encephalitis, gliosis and perivascular cuffing. IHC detects H5N1 related-viral antigens in alveolar macrophages, neurons and glial cells. Epithelial cells, lining the bronchi and small intestine, are also H5N1 immunopositive [266,298,300].

#### 11.2.3. Domestic and Wild Canids (Dogs and Foxes)

The susceptibility of canids to HPAIVs was confirmed in experimental infections in dogs and foxes [134,309,310]. Natural infection in dogs [298,307] and foxes was recorded and some cases were described in foxes in Belgium [193,311]. Fox is a predator/scavenger animal and fox infection may occur after eating muscle of hunted H5N1 positive wild birds or infected body carcasses.

Their infection may result in a variation in outcomes from asymptomatic [307] to subclinical infection or serious illness with respiratory and neurological signs [193,298,311].

Fox gross pulmonary lesions ranged from edema to diffuse parenchymal consolidation, while no gross lesion in CNS was recorded. Histologically, a mild-to-severe necrotizing interstitial pneumonia with fibrin, neutrophil granulocytes infiltration and occasional type II pneumocyte hyperplasia were observed, while a severe meningoencephalitis, with infiltrates of lymphocytes, plasma cells, macrophages, rare neutrophils and reactive astrocytes, was recognized in the CNS [193,311]. Dogs are susceptible to H5N1 infection, always with an asymptomatic course. At necropsy, a multi-organ congestion (lung, spleen, liver and kidney) and pulmonary oedema were recorded, while no gross lesions were observed in the CNS. Histologically, lungs showed alveolar oedema and interstitial pneumonia. Hepatocytes necrosis and nephritis/tubular nephrosis were the most relevant features in other organs. IHC pointed out the presence of the H5N1 virus-related antigen in alveolar epithelial cells, hepatocytes and renal tubular epithelium [298].

#### 11.2.4. Ruminants (Cattle)

In naturally occurring cases of HPAIV H5N1 infection in cattle, a subclinical course or low-grade respiratory involvement under field conditions with no-to-minimal gross pulmonary lesions was frequently recorded. In experimentally infected cattle, at necropsy, an interstitial pneumonia was recognized.

Histopathology revealed interstitial pneumonia, often characterized by fibrin deposition in the alveolar spaces and moderate thickening of the alveolar septa due to cellular infiltration of neutrophils, macrophages, and lymphocytes. Peribronchiolar inflammatory cellular infiltration was mild and confined to the walls of the affected bronchioles.

Gastrointestinal findings were recognized as small abomasal ulcers and linear erosions of the intestinal mucosa but were uncommon.

No consistent gross pathological changes were detected in the central nervous system, myocardium, or parenchymatous organs such as the liver, kidney, pancreas or spleen [128,283].

In the mammary glands of dairy cows, experimentally infected with H5N1 clade 2.3.4.4b virus, a marked degeneration of epithelial milk-secreting cells was recorded. The H5N1 clade 2.3.4.4b viral cytopathic effect was characterized by epithelial cell swelling, vacuolization and impaired milk secreting function. Histopathology showed a disruption of the alveolar architecture, infiltration of inflammatory cells and necrosis. Epithelial milk-secreting cells were positive for H5N1 antigen. These new pathological findings expand tissue tropism of H5N1 as well as a novel route for H5N1 virus transmission with zoonotic risk [257,283].

HPAIV A (H5N1) clade 2.3.4.4b infection was confirmed in sheep but no gross or histopathological lesions were described [312].

#### 11.2.5. Goats

Goat presents marked serological reactivity toward HA antigens derived from AIVs, including those associated with HPAIV subtype H5. In HPAIV H5 infected goats, a significant humoral immune response was detected, that supports the natural species susceptibility to avian-origin influenza A H5 virus. No gross or histopathological lesions were described [313].

#### 11.2.6. Camelids (Dromedary Camels and Alpacas)

Antibodies against influenza A H1N1 and H3N2 viruses and an influenza A H1N1 pdm09–like virus were detected in a dromedary camel (*Camelus dromedarius*) in Nigeria. No gross or histopathological lesions were described [314].

H5N1 virus infection was confirmed in 4 out of 18 alpacas (*Lama pacos*) that had been epidemiologically linked to poultry premises affected by HPAI in Idaho (USDA). No gross or histopathological lesions were described [315].

In alpacas, an immune response was also recorded following H5N1 immunoprophylactic antigen exposure. No gross or histopathological lesions were described [316].

#### 11.2.7. Equids (Donkeys and Horses)

Equids are susceptible to infection by AIV H5N1. Abdel-Moneim and co-workers described an outbreak of influenza in donkeys in Egypt in 2009; they isolated IAV H5N1 from nasal swabs and demonstrated that about 26% donkeys sampled had been infected. No gross or histopathological lesions were described [317]. Mongolian horses tested serologically positive of H5N1. No gross or histopathological lesions were described [318].

#### 11.2.8. Swine

Pigs experimentally exposed to HPAIV H5N1 clade 2.3.4.4b through their nostril and digestive tract showed limited virus replication and an asymptomatic course, but 100% of infected pigs seroconverted [319,320,321].

Experimental infection caused interstitial pneumonia, with necrotizing bronchiolitis and high titers of virus in the lower respiratory tract. Infected pigs shed limited amount of virus with no risk of pig-to-pig transmission [320,322].

#### 11.2.9. Wild Animals

H5N1 clade 2.3.4.4b viruses have a marked capacity of cross-species transmission, being able to infect phylogenetically diverse mammalian taxa, including terrestrial, semi-aquatic, and marine species. This broad host adaptability reflects the virus enhanced molecular plasticity and ability to overcome host-specific cellular and immunological barriers.

In North America, numerous free-ranging mammalian species have been reported as naturally infected by HPAIV H5N1, highlighting the broad host range and cross-species transmission. A wide taxonomic spectrum, including terrestrial carnivores and mesocarnivores frequently exposed to infected avian prey or carcasses, becomes infected.

Foxes such as red foxes (*Vulpes vulpes*), gray foxes (*Urocyon cinereoargenteus*) are notoriously susceptible to H5N1 viral infection, but other terrestrial carnivore infections have been positively tested: striped skunks (*Mephitis mephitis*), American mink (*Neovison vison*), raccoons (*Procyon lotor*), coyotes (*Canis latrans*), bobcats (*Lynx rufus*) and Virginia opossums (*Didelphis virginiana*). Larger carnivores and omnivores such as black bears (*Ursus americanus*), polar bears (*Ursus maritimus*), grizzly bears (*Ursus arctos horribilis*) and mountain lions (*Puma concolor*) have also tested positive for H5N1 virus [157].

In wild mammals naturally infected with HPAIV H5N1, the distribution, extent and severity of lesions were related to interspecies variability, reflecting differences in host susceptibility, immune response, and viral tissue tropism.

The most consistent and prominent gross pathological features primarily involve the pulmonary vascular system, characterized by pulmonary edema, vascular congestion and focal parenchymal hemorrhages.

In the lungs, a necrotizing interstitial pneumonia represents the more important diagnostic finding. At histopathology, alveolar septal necrosis, intra-alveolar edema and mononuclear inflammatory cell infiltration, fibrin exudation and hyaline membrane formation in severe cases were detected.

Regarding the CNS, focal-to-multifocal malacia of the brain parenchyma due to hemorrhages may occur, although the severity and distribution of gross and histologic lesions may vary. At histopathology, brain represents the most consistently and severely damaged organ. Brain lesions were virus-associated and revealed as lymphoplasmacytic perivascular cuffing, occasionally cellular-mixed with neutrophilic and eosinophilic granulocytes. Neuronal necrosis, microgliosis and astrocytic hypertrophy were also present.

In the heart, myocardial degeneration and necrosis were also frequently recorded, sometimes accompanied by focal mineralization of degenerated myofibers.

Other organs were less frequently involved. The most important lesions were an acute random hepatic necrosis and lymphoid depletion or necrosis affecting the spleen Malpighian germinal centers as well as the regional lymph nodes. Multifocal necrosis was recognized in the kidney, pancreas, and gastrointestinal tract, due to systemic viral dissemination and multiorgan involvement in advanced infection and progression [32,157,323].

#### 11.2.10. Polar Bears

At necropsy, skin ulcers, banding, eye and oral commissure were recognized at external body examination. In cavitary organs, congestion and edema were the most evident features in liver, lungs and brain. In the pericardium sac, a cavitary fluid collection was observed. At histopathology, granulocytic and mononuclear meningoencephalitis, microgliosis, neuronal necrosis, neuronophagia, vasculitis and cerebral malacia were described [158].

#### 11.2.11. Mustelidae (Male Fishers and Mink)

HPAIV H5N1 clade 2.3.4.4b infection included male fishers (*Pekania pennanti*), which, like other mustelids (viverrids), may serve as sentinel species due to their scavenging and predatory habits that facilitate exposure to infected avifauna [157].

Agüero and co-workers (2023) reported a HPAIV A H5N1 virus infection in a farmed mink in Spain, providing information on SA receptors like those of human influenza A, but no gross pathology and histopathology were reported [214].

#### 11.2.12. Aquatic Mammals

H5N1 viral infection was recognized in semi-aquatic and marine species such as American river otters (*Lontra canadensis*), harbor seals (*Phoca vitulina*) and gray seals (*Halichoerus grypus*), as well as cetaceans such as the bottlenose dolphin (*Tursiops truncatus*). South American sea lions (*Otaria byronia*) and southern elephant seals (*Mirounga leonina*) were the most affected marine mammals, but smaller epizootics in South American fur seals (*Arctocephalus australis*), otters (*Lontra* spp.) and cetaceans have also been linked to HPAIV H5.

Clinical signs in seals and sea lions were mainly neurologic, characterized by tremors, convulsions, and paralysis. On post-mortem examination, there were whitish secretions in the upper respiratory tract and congestion of the lungs and brain [38,153,154,155,156].

#### 11.2.13. Cetaceans (Bottlenose Dolphins)

In bottlenose dolphin (*Tursiops truncatus*), at necropsy, a severe pharyngeal mucosa erosion and lymphoadenomegaly were the most prominent lesions compatible with H5N1 virus infection. At histopathology, mononuclear meningoencephalitis (lymphocytes and histiocytes), neuronal necrosis and encephalic leukomalacia were recognized. Blood meningeal and brain vessels were surrounded by perivascular mononuclear cuffing and multifocal acute hemorrhages. A mild multifocal gliosis was also recorded [96,156,324,325,326,327].

#### 11.2.14. Flying Mammals (Bats)

Recently, it was reported that the antigen of H5N1 virus was detected in tissues samples (trachea, kidney, liver, lungs, and brains) of fruit bats. The H5N1 clade 2.3.2.1a virus is closely related to sequences from house crows and was detected in Indian flying foxes (*Pteropus medius*), in Bangladesh. No description was reported for the clinical course or gross pathology and histopathology [159].

## 12. Regulatory Basis

### 12.1. International Legislation

According to Art. 3 of the Sanitary and Phytosanitary Measures (SPS) Agreements [328], World Trade Organization (WTO) member states (accounting for about 98% of world trade) must comply, when establishing trade agreements or resolving disputes, with objective scientific standards established by specific reference authorities, specifically the *Codex Alimentarius* Commission [329] (an authority established by the FAO and the WHO), and the OIE (*Office International des Epizooties*)-WOAH [330].

The health standards for defining AI-free territory and for health guarantees associated with international animal movements are contained in the OIE Terrestrial Animal Code (Chapter 10.4), while the accredited diagnostic and surveillance methods are contained in the Terrestrial Animal Manual (Chapter 3.3.4) [331].

### 12.2. European Legislation: Regulation (EU) 2016/429: Animal Health Legislation

As of April 2021, Regulation (EU) 2016/429 [332] repealed the previous Directive 2005/94/EC, which regulated AI, replacing it and all other previous vertical animal health legislation.

Regulation 2016/429, also known as “Animal Health Law”, aims to implement the One-Health principle for the unified management of animal health with human health and the definitive harmonization of EU regulations with WOAH standards.

### 12.3. Delegated and Implementing Acts

The full implementation of the general principles contained in Regulation (EU) 2016/429 is entrusted to a series of delegated and implementing regulations, approved by the European Commission and subject to periodic updates to adapt the European standard to new developments arising from scientific progress and the epidemiological evolution of infectious diseases:

(1) Implementing Regulation (EU) 2018/1882: categorization of listed diseases. HPAI is classified as a Category A + D + E disease (a listed disease which does not normally occur in the Union and which, upon detection, requires the immediate adoption of eradication measures), while LPAI is classified as a Category D + E disease (a disease for which measures are necessary to prevent its spread due to its entry into the Union or movement between Member States and for which surveillance within the Union is necessary) [333];
(2) Delegated Regulation (EU) 2020/687: rules on the prevention and control of certain listed diseases [334];
(3) Delegated Regulation (EU) 2020/689: surveillance, eradication programs, and disease-free status for certain diseases [335];
(4) Implementing Regulation (EU) 2020/690: territorial surveillance areas for certain listed diseases [336];
(5) Delegated Regulation (EU) 2020/2002: notification of listed diseases in the Union [337];
(6) Implementing Regulation (EU) 2021/620 and subsequent amendments: approval of the disease-free status and eradication programs of Member States and certain zones or compartments thereof [338];
(7) Delegated Regulation (EU) 2023/361: rules for the use of certain veterinary medicinal products for the prevention and control of certain listed diseases [339].

### 12.4. Italian Legislation

(1) Legislative Decree 05/08/2022 nr. 136: national legislation on the prevention and control of transmissible animal disease [340];
(2) Ministerial Decree 30/05/2023: application methods of biosecurity measures in poultry farms [341].

It is important to underline that there are no substantial differences between the measures of the new Animal Health Regulation and the previous Directive 2005/94/EC regarding HPAI, while the approach to LPAI (see below) has radically changed.

### 12.5. National Surveillance Plan

The national AI surveillance plan [342] has the following objectives:

(1) early detection of HPAI in poultry;
(2) early detection of HPAI in wild birds, providing:
  - an early warning system for the possible introduction of HPAI into domestic poultry;
  - information for assessing the risk of virus spread;
(3) detection of HPAI in poultry species that generally do not show significant clinical signs;
(4) detection of circulating LPAIVs, particularly in areas with high livestock density, considering their potential to mutate into HPAIVs;
(5) contribution to increasing knowledge on HPAIVs and LPAIVs that pose a potential zoonotic risk.

In compliance with the requirements of Regulation (EU) 2020/689 [335], Annex 2, the plan includes a differentiation of sampling activities and therefore provides for risk-based surveillance (RBS), which considers the following factors:

(1) location of poultry farms near wetlands or in areas with a high density of migratory wild birds;
(2) location in densely populated poultry areas (DPPAs);
(3) farms within the wintering areas of mallard ducks;
(4) structural and management characteristics of the poultry production system;
(5) current and historical epidemiological situation;
(6) trade flow and type;
(7) species and production type (long-lived, multi-age, multi-species poultry) and biosecurity measures for poultry farms;
(8) biosecurity measures for ordinary farms of protected species;
(9) presence of free-range poultry farms and/or farms where poultry may come into contact with wild birds;
(10) risk assessment and scientific opinions issued by the National Reference Centre for Avian Influenza and Newcastle Disease.

“High-risk” provinces were therefore identified, requiring high-frequency monitoring, and “medium-risk” provinces required less frequent monitoring.

The provinces at high risk of introduction and spread in Italy are:

(1) Emilia-Romagna: Bologna, Ferrara, Forlì-Cesena, and Ravenna;
(2) Lombardy: Bergamo, Brescia, Cremona, and Mantua;
(3) Piedmont: Cuneo;
(4) Veneto: Padua, Rovigo, Venice, Verona, and Vicenza.

The provinces at medium risk are:

(1) Friuli-Venezia-Giulia: Pordenone and Udine;
(2) Lazio: Viterbo;
(3) Umbria: Perugia and Terni;
(4) Veneto: Treviso.

The remaining part of the national territory has been classified as “low-risk”.

In the provinces identified as high-risk, active surveillance involves all commercial poultry farms with ≥250 birds. In the provinces at medium risk, surveillance is conducted on a sample basis.

The farm monitoring plan involves a two-stage sampling (a sample of farms and a sample of animals from each selected farm), differentiated by species.

In high-risk provinces, the serological sampling objectives regarding the number of farms to be inspected and samples to be collected from each farm are analytically specified within the plan using specific tables for each farmed species, region, and province.

### 12.6. Sampling Scheme for Active Risk-Based Surveillance

In the provinces identified as being at high risk, all poultry farms with ≥250 birds must be sampled, according to the following procedures:

(1) meat turkeys: serological sampling from 5 birds per barn, with a minimum of 10 birds per farm, once a year;
(2) breeding quails: on-farm virological sampling of at least 20 birds every six months;
(3) fattening and breeding ducks and geese: virological sampling (cloacal swabs on individual birds and/or fresh feces pools) every 6 months from 5 birds per production unit, with a minimum of 10 birds per farm;
(4) breeding farms (except ducks, geese, and quails) and laying hens for the production of eggs for consumption: annual serological sampling from 5 birds per production unit, with a minimum of 10 birds per farm;
(5) other meat birds (excluding broilers and quails): annual serological sampling of at least 10 birds per farm;
(6) game: 5 blood samples per aviary every 6 months (10 samples if the farm consists of a single aviary);
(7) ratites: annual serological sampling of at least 10 animals per farm.

In areas classified as having a medium risk of introduction and spread of AIVs, the following must be subjected to active surveillance:

(1) fattening and breeding turkeys and breeding chickens;
(2) laying hens, both indoors and free-range;
(3) breeding game birds;
(4) fattening and breeding ducks and geese.

For each of the above-mentioned production categories (excluding ducks and geese for meat and breeding), the number of establishments to be sampled annually is based on a representative sampling scheme (with a 95% probability of identifying at least one positive animal, assuming a prevalence of 5%).

On each farm, serological samples must be collected from a minimum of 10 randomly selected animals (with a 95% probability of identifying at least one positive animal, assuming a prevalence of 30%). If the farm has more than one shed, samples will be taken from 5 animals per unit.

For meat and breeding ducks and geese, virological samples (cloacal swabs and/or fresh fecal pools) must be collected every 6 months from 5 animals per production unit, with a minimum of 10 samples per farm. If the farm has only one shed, 10 animals must be sampled.

Regarding rural farms, a total of 500 farms must be inspected in high- and medium-risk areas, with inspections taking place every 6 months (in spring and autumn, coinciding with the migratory phases of wild birds). At least 10 animals must be sampled for virological testing on each farm.

In the remaining national territory, classified as low-risk, nursery farms must be subjected to serological sampling: at least 5 animals per production unit, with a minimum of 10 animals per farm and a maximum of 20 animals. If ducklings are present, cloacal swabs or fresh feces must be collected for virological testing, with the same number required for serum samples.

Samples must be taken monthly for nursery farms accredited for extra-regional trade and those authorized to participate in fairs or markets and quarterly for the remaining farms.

The selection of animals to be sampled must be representative and based on the following epidemiological priority criteria:

(1) protected species;
(2) older animals relative to their category;
(3) free-range animals;
(4) animals returning from fairs, exhibitions, and markets;
(5) other categories deemed significant based on the official veterinarian’s assessment.

### 12.7. Virological Surveillance During the High-Risk Season

In light of the changing epidemiological conditions, an additional surveillance plan is planned to be carried out between September 15 and March 15 in the high-risk provinces of the Veneto and Lombardy regions, according to the following guidelines:

(1) meat turkeys, pullets, and laying hens: a number of farms must be selected on a sample basis to exclude the circulation of influenza viruses with an inter-farm prevalence of 3% and a 95% confidence level;
(2) broilers: all farms having birds between 37 and 44 days of age must be tested.

Sampling must be conducted every 15 days. At least 10 tracheal swabs must be collected from each farm from birds that died of natural causes on the day of sampling (or, in their absence, those that died in the days immediately preceding the day of sampling) and/or from symptomatic or moribund birds.

### 12.8. Wild Bird Surveillance Program

The wild bird surveillance plan essentially involves passive surveillance based on virological testing of birds found dead or symptomatic.

Surveillance primarily concerns wetlands located along wild bird migration routes, targeting a population primarily composed of aquatic species belonging to species targeted for AI.

The species involved are the following:

(1) *Podicipedidae* (grebes);
(2) raptors (diurnal and nocturnal);
(3) *Ardeidae* (herons);
(4) *Anatidae* (ducks, geese, and swans);
(5) *Rallidae* (coot, moorhen, purple gallinule, and others);
(6) *Recurvirostridae* (avocet and black-winged stilt);
(7) *Charadridae* (plovers and lapwings);
(8) *Scolopacidae* (waders);
(9) *Laridae* (gulls);
(10) *Sterninae* (terns).

### 12.9. Eradication Measures in the Event of an Outbreak

#### 12.9.1. Definition of Suspected and Confirmed Disease

In Delegated Regulation (EU) 2020/689 (Art. 9, Annex 1) [335], the definitions of suspected and confirmed disease, while remaining substantially unchanged from previous regulations, are formulated as follows:

(1) suspected case of HPAI (or LPAI): an animal or group of animals showing clinical symptoms or anatomo-pathological lesions indicative of the presence of the disease, that has reacted positively to a laboratory test, or is epidemiologically related to a confirmed case;
(2) confirmed case of HPAI (or LPAI): an animal or group of animals that tests positive for virus isolation or nucleic acid testing (RT-PCR), or for which clinical symptoms and/or epidemiologically correlate with a positive indirect diagnostic test (serological test).

An HPAIV is defined as such, regardless of its subtype, if it has an intravenous pathogenicity index (IVPI) greater than 1.2, or if it belongs to the H5 or H7 subtypes and possesses a multiple basic amino acid sequence at the hemagglutinin cleavage site.

An LPAIV is considered such if it belongs to the H5 or H7 subtypes but lacks the above characteristics. The definition of AI caused by HP viruses coincides with the disease formerly known as fowl plague [332,333].

#### 12.9.2. Measures in Case of Suspected HPAI

Suspicion of HPAI may therefore arise from clinical, laboratory, or epidemiological evidence. In case of suspicion, the following measures apply:

(1) notification of a suspected case (in Italy, through the “Sistema Informativo Malattie Animali”, SIMAN, animal disease information system);
(2) seizure of the farm;
(3) checking of farm and movement records;
(4) conducting an epidemiological investigation;
(5) clinical visits to all production units;
(6) standard collection of biological samples and delivery to the laboratory [332,333].

#### 12.9.3. Measures in Case of HPAI Confirmation

The measures in case of confirmed cases of HPAI are the following:

(1) notification of a confirmed case (SIMAN);
(2) seizure and isolation measures for the affected farm from the time of suspicion, extended to all animal species bred in contact with poultry;
(3) completion of the epidemiological investigation and tracing of epidemiologically related farms;
(4) culling of all poultry (stamping-out);
(5) destruction of carcasses, eggs, and any other potentially contaminated material;
(6) disinfection of the site under official supervision;
(7) management of the emergency standardized by the operating manual;
(8) differentiation of measures for LPAI outbreaks (see below) [332,333].

#### 12.9.4. Exceptions

Exceptions from stamping-out measures are possible for epidemiologically distinct farm units (Art. 13.1); in these cases, the competent authority must conduct a preliminary risk assessment and, if necessary, laboratory sampling, demonstrating complete separation of the epidemiological units considered at least for the entire monitoring period referred to in Annex 2. Further exceptions (Art. 13.2) are provided for confined establishments, animals kept for scientific purposes or for the conservation of protected species, and animals of rare breeds or of high genetic value. In all these cases, the granting of exceptions is subject to a risk assessment and the implementation of biosecurity guarantees and surveillance measures to prevent the spread of the disease [332,333].

### 12.10. Extension of Culling Measures

Flexibility in applying stamping-out measures is also envisaged in an expansive sense: specifically, it is possible to extend stamping-out measures to epidemiologically related farms (Regulation (EC) 2020/687, Arts. 17 and 18) [334] or to animals of unlisted species (e.g., pigs—Art. 14) or wild animals, always based on a risk analysis that establishes the need for such measures to combat the spread of the disease.

If pigs are present in the outbreak facility, the preparatory actions for risk assessment should be as follows:

(1) nasal swab sampling from 60 animals or all animals present, if the total is less, on the day of culling;
(2) serological testing on 60 animals, conducted from 14 to 28 days after culling.

If the results of laboratory tests and the epidemiological investigation suggest a serious risk of disease spread, stamping-out measures must be applied.

In the event of an HPAI outbreak on a farm raising other animal species, the competent authority, based on the results of the risk analysis, may extend the mandatory culling measures to all animal species present [332,333].

### 12.11. Territorial Restrictions

#### 12.11.1. Protection Zone

A protection zone (PZ) must be established around the outbreak, with a minimum radius of 3 km.

The main obligations are:

(1) carry out a census and clinical examination as soon as possible on all poultry farms;
(2) report any increase in mortality to the official veterinarian;
(3) record farm visits;
(4) lock all reared birds inside their sheds and adopt measures to avoid contact with wild birds.

The main prohibitions are:

(1) movement of live poultry, meat from slaughterhouses located within the PZ, eggs, and carcasses;
(2) slaughter;
(3) outdoor housing;
(4) fairs and markets;
(5) outdoor storage of manure.

The exceptions to the movement ban apply to:

(1) the immediate slaughter; in this case, the meat obtained, in addition to compliance with specific safeguards (clinical examination within 24 h prior to departure), is subject to special branding and heat treatment before release for consumption; the heat treatment is not necessary if the meat is intended for the national market only and is subjected to the special marking referred to in Annex 9.
(2) the movement of hatching and table eggs, day-old chicks and pullets, and carcasses intended for destruction.

In case of revocation, laboratory tests must be performed on the samples required by the operating manual (5 died animals + 20 tracheal swabs + 20 cloacal swabs + 20 blood serum samples), at least at 21 days after disinfection of the outbreak site, in all farms within the PZ. After the PZ is revoked, the surveillance zone (SZ) measures remain in effect until its revocation [332,333].

#### 12.11.2. Surveillance Zone

A surveillance zone (SZ) must be established around the protection zone, with a minimum radius of 10 km around the outbreak.

The main obligations are:

(1) census of poultry farms;
(2) reporting of any increase in mortality to the official veterinarian;
(3) recording of farm visits;
(4) locking of all the birds reared inside the sheds and adopting measures to avoid contact with wild animals.

The main prohibition is the movement of poultry, pullets, day-old chicks, and eggs, unless authorized by the competent authority.

Exceptions to the movement ban are permitted, subject to specific health guarantees for:

(1) immediate slaughter;
(2) the movement of pullets, day-old chicks, hatching eggs, table eggs, egg products, and eggs intended for destruction.

Revocation: at least 30 days after disinfection of the outbreak [332,333].

#### 12.11.3. Further Restricted Zones

The competent authority may establish additional restricted zones (RZs), concentric with the PZs and SZs, in which the same restrictions as those established for the SZs apply.

In December 2024, in response to new H5N1 outbreaks in Italy, the Ministry of Health issued a management order introducing additional RZs in the most affected areas of the Lombardy, Veneto, Emilia-Romagna, and Friuli-Venezia Giulia regions. This order, valid until 31 January 2025, provided for enhanced surveillance and containment of the spread of the virus in the aforementioned areas, considered to have a high density of poultry farms and affected by the migration of wild birds [332,333].

#### 12.11.4. Repopulation of Farms

In farms affected by an outbreak, subsequent repopulation is subject to a series of restrictions:

(1) verification and favorable assessment of biosecurity conditions;
(2) a minimum 21-day period following post-culling disinfection; the sanitary vacuum continues for the entire duration of the PZs or surveillance measures;
(3) collection of biological samples from the introduced animals [332,333].

### 12.12. Vaccination Plans

Vaccination for AI is generally prohibited. As an exception to the general ban, EU legislation allows for the implementation of emergency vaccination or preventive vaccination plans based on risk analysis (EU Regulation 2016/429, Arts. 43 and 69) [332].

Delegated Regulation (EU) 2023/361 [339] regulates the adoption of vaccination measures in the event of the introduction of a Category A disease into the European territory. Following the outbreak, the competent authority may decide to carry out (Art. 7):

(1) emergency suppressive vaccination within disease outbreaks;
(2) emergency prophylactic vaccination in establishments not affected by the disease within RZs;
(3) preventive vaccination in unaffected geographical areas.

AI vaccination is based on the so-called “differentiation of infected animals from vaccinated animals” (DIVA) strategy, which involves the use of a killed vaccine containing the same HA as the field virus but a different NA. This allows for laboratory differentiation of any positive serological results found during surveillance activities [241].

The application of vaccination strategies, both emergency and preventive, has often proven indispensable for controlling the epidemiological situation in the densely populated areas of north-eastern Italy.

### 12.13. Biosecurity Measures

Minimum biosecurity requirements for AI are specified in the Ministerial Decree of 30 May 2023—Application of Biosecurity Measures in Poultry Farms [334].

Annex A divides facilities into ordinary farms with a capacity of up to 250 birds (Section 2) or more than 250 birds (Section 3); additional measures are also provided for outdoor farms (Section 3.c.).

Additional biosecurity requirements apply to handling (Section 4), minimum distances between facilities (Section 5), cleaning and disinfection (Section 6), sanitary vacuum, management of dead animals and litter, weaning facilities, fairs and markets, and packing centers.

Some of the main structural and management requirements are:

(1) barriers at the farm entrance to prevent unauthorized entry of people and vehicles;
(2) filter areas for employee changing rooms;
(3) loading/unloading areas for animals/materials and disinfection areas for vehicles;
(4) protection of animal feed storage areas;
(5) washable and disinfectable floors, walls, and equipment;
(6) sparrow-proof mesh on entrances (except warehouses with external platforms);
(7) prohibition of entry to outsiders;
(8) use of disposable or washable and disinfectable clothing (shoes);
(9) registration of entry and exit of personnel, animals, equipment, and vehicles;
(10) rodent control and insect control programs;
(11) prohibition of keeping birds by farm personnel;
(12) periodic disinfection;
(13) storage of carcasses in cold storage and disposal by authorized companies.

Annex B lists the criteria for identifying high-risk areas for AI by the regions divided into Zones A and B and the related restrictions to be applied in each zone.

## 13. Conclusions

The worldwide spread of AIVs across different animal species, environments and countries highlights a clear One-Health challenge. Global AI transmission risks highlight the need to strictly adopt and enforce national and international rules (WOAH and WHO guidance), surveillance and reporting obligations, movement controls, culling and vaccination policies, and harmonized food safety standards to manage these hazards.

As AVs responsible for new outbreaks can change over time and constitute a threat for new spill-over events, and potentially for new pandemics, continuous evaluation and updating of vaccine seed strains for vaccine adaptation must be considered for protection against emergent variant field strains.

Many animal species belonging to the classes Reptilia, Amphibia and Pisces are only minor dead-end hosts. Semi-aquatic mammals are not primary, long-term reservoirs like wild waterfowl, but they play an important role as spillover hosts and, in specific contexts, as amplification hosts, particularly under conditions of intensive farming of semi-aquatic carnivores (especially mink), in rehabilitation, in captive facilities (e.g., wildlife rescue centers and zoos housing semi-aquatic mammals) and within groups exposed to heavily contaminated environments at the water–land interface (e.g., sea otter families, muskrat colonies). Ferrets, dogs, and cats can be infected by both AI strains and human influenza strains under experimental conditions and are often used as models of human infection (especially ferrets), but their real-world importance as “mixing vessels” for influenza viruses appears to be limited and is not comparable with that of pigs. Horses are not recognized as important mixing vessels, and their public-health and food-chain relevance is minimal.

Key mixing vessels such as pigs and certain domestic birds (e.g., quails) drive viral adaptation at the animal–human interface. Dairy cows are not currently considered “mixing vessels” like pigs; instead, they mainly act as spillover or amplification hosts.

Therefore, genomic monitoring of influenza viruses in mammals is pivotal to evaluate the genome modifications occurring in viral strains or subtypes responsible for new outbreaks and disease diffusion. Raw milk from AIV-infected dairy cattle can contain H5N1 virus and plays an important role in cow-to-calf transmission through nursing. Pasteurized milk and derived products (e.g., cheese, yogurt, butter) are considered safe for consumers when pasteurization is correctly performed, as well as seasoned cheese (ripening involves low pH, salt, competing microflora, reduced water activity and time). In addition, updated and improved biosecurity regulations are of great importance for dairy and mixed breeding in order to minimize the chance of inter-species viral spread.

Strict hygiene in livestock production, food industries, intensive mink farming, zoo facilities, aquatic–terrestrial interface, and the use of personal protective equipment (gloves, masks, eye protection) by farm workers and veterinarians are essential to reduce zoonotic and food-borne transmission. Broadly, these coordinated, cross-sectoral interventions are critical to protect animal health, safeguard the integrity of the food chain and consumers, and mitigate the risk of future zoonotic AI outbreaks and related global health emergencies.

## Figures and Tables

**Table 1:** Distribution of sialome receptors (α2,3-linked and α2,6-linked sialic acid [SA]) in most susceptible tissues, excluding the central nervous system (CNS), of human and domestic mammalian species and relevance for HPAIV infection.

Mammalian Species/Group	Tissue/System	α2,3-Linked SA (Avian-Type)	α2,6-Linked SA (Human-Type)	Relevance for HPAIV Infection/Tropism	References
Human	Upper respiratory tract (nasal, trachea)	Present but generally lower density than α2,6-SA	Predominant on ciliated and non-ciliated epithelial cells	Favors binding and replication of human-adapted influenza viruses; limited but possible binding of AIV through α2,3-SA; key site for zoonotic spillover events	[14]
Human	Lower respiratory tract (bronchioles, alveoli)	Abundant on type 2 pneumocytes and some non-ciliated cells	Present on selected epithelial and endothelial cells	Co-expression allows for infection by both avian and human strains; deep lung α2,3-SA associated with severe AIV pneumonia and ARDS	[14]
Human	Intestine	Detected on intestinal epithelial cells and goblet cells	Detected on subsets of enterocytes	Possible enteric involvement in severe AIV infection; fecal shedding reported in some H5N1 infection cases, but respiratory route remains dominant	[14,48]
Swine (pig)	Respiratory tract	Mixed expression of α2,3-SA and α2,6-SA on tracheal and bronchial epithelium	Mixed expression on respiratory epithelium and submucosal glands	Dual receptor repertoire underlies “mixing vessel” role, allowing for co-infection and reassortment between avian and human influenza viruses	[121]
Swine (pig)	Intestine, other organs	Both α2,3-SA and α2,6-SA are present in multiple organs, including intestinal epithelium and vascular endothelium	Co-expressed in many tissues	Broad tissue distribution favors systemic spread of adapted strains and facilitates generation of novel reassortants with zoonotic potential	[121]
Bovine (dairy cow)	Mammary gland (lactating)	Mammary epithelium rich in α2,3-SA	Limited data; minor or focal α2,6-SA reported	Supports H5N1 clade 2.3.4.4b viral replication in lactating-secreting epithelial cells; associated with viral mastitis and shedding in raw milk, enabling intra- and inter-species transmission	[58,127,128,129,130,131]
Bovine (beef cattle)	Respiratory tract	Present but less dominant than α2,6-SA	Respiratory epithelium rich in α2,6-SA	Favors infection with more mammal-adapted or reassortant strains; AIV detection in respiratory tissues documented in experimental and field cases	[129,132]
Bovine (dairy and beef)	Muscle (meat)	Detected in muscle cells by some assays	Detected in muscle cells	Virus can be detected in muscle of infected animals; foodborne transmission risk considered low if proper cooking is applied but relevant for surveillance in meat products	[129,132]
Feline (cat)	Respiratory tract	α2,3-SA present on respiratory epithelium	α2,6-SA also expressed in the respiratory tract	Mixed receptors allow for infection by both avian- and human-like viruses; cats are susceptible to H5N1 strain and other AIVs and can develop severe respiratory and systemic disease	[133]
Canine (dog)	Respiratory tract	α2,3-SA localized on epithelial cells of nasal, tracheal and bronchial mucosa (immunohistochemistry [IHC] and lectin studies)	α2,6-SA present but less extensively mapped	Dogs can be infected by avian-origin influenza viruses (e.g., H3N2, H5N1); α2,3-SA expression in upper airways supports susceptibility to AIV	[134,135]
Equine (horse)	Upper respiratory tract (nasal, tracheal)	Predominantly “duck-type” α2,3-SA (SA–α2,3–Gal–β–GalNAc) on epithelial cells lining nasal and tracheal mucosa	Limited α2,6-SA reported in upper airways	Avian-type receptor dominance suggests theoretical susceptibility to AIV; natural infection is rare, but equine influenza ecology may intersect with avian viruses in some settings	[136,137]
Rodents (e.g., mouse and ferret as models)	Respiratory tract	Species-dependent; laboratory mice often show substantial α2,3-SA in lower airways; ferrets express both α2,3- and α2,6-SA similar to humans	Ferrets: robust α2,6-SA in upper airways Mice: more restricted α2,6-SA pattern	Ferrets are a key model for both human and avian influenza because of their mixed sialome; mice are useful for H5N1 viral pathogenesis, though their receptor distribution differs from humans	[105,106,107]

**Table 2:** Distribution of sialome receptors (α2,3-linked and α2,6-linked SA) in the CNS of different mammals (human and domestic) and relevance for HPAIV infection.

Mammalian Species/Group	Tissue/System	α2,3-Linked SA (Avian-Type)	α2,6-Linked SA (Human-Type)	Relevance for HPAIV Infection/Tropism	References
Human	CNS (neurons, glia, endothelium, ependyma)	Both α2,3-SA and α2,6-SA widely expressed on neurons, glial cells, endothelial and ependymal cells	Co-expressed with α2,3-SA in multiple CNS cell types	Supports potential neurotropism and neuroinvasion of AIVs (e.g., H5N1) once the blood–brain barrier is compromised; may contribute to neurological signs in severe infections	[138,139]
Swine (pig)	CNS	α2,3-SA detected on neurons	α2,6-SA detected mainly on vascular endothelial cells	CNS sialome pattern is compatible with potential neurotropic infection by AIVs, particularly highly pathogenic H5N1 strains	[121]
Bovine (dairy and beef)	CNS (cerebrum)	α2,3-SA present in neuronal and glial populations	α2,6-SA also present in cerebrum	Compatible with neurotropic infection by H5N1 virus; CNS involvement documented in some experimental infections	[132]
Feline (cat)	CNS (cerebellum, other regions)	α2,3-SA detected in cerebellar tissue	α2,6-SA co-expressed in cerebellum	CNS expression supports potential neurotropism of H5N1 virus in cats, with reported neurologic signs and CNS lesions in natural and experimental infections	[133]
Canine (dog)	CNS and vascular endothelium	α2,3-SA on vascular endothelial cells in CNS and other organs	α2,6-SA co-localized on vascular endothelial cells	Co-expression may allow for systemic and neurotropic infection by highly pathogenic AIV strains under certain conditions	[135]

**Table 3:** Distribution of sialome receptors (α2,3-linked and α2,6-linked SA) in tissues of selected marine mammals and flying mammals (Chiroptera) and relevance for AIV infection.

Species/Group	Tissue/System	α2,3-Linked SA (Avian-Type)	α2,6-Linked SA (Human-Type)	Relevance for AIV Infection/Tropism	References
Seals and sea lions (pinnipeds)	Upper respiratory tract (nasal cavity, trachea)	Abundant on respiratory epithelial cells; strong binding of α2,3-specific lectins and avian-like HA in several species	Detected at lower density on some epithelial and submucosal gland cells	Dominant α2,3-SA in upper airways supports susceptibility to avian-origin AIVs (e.g., H3N8, H10N7, H5N8); multiple outbreaks with respiratory disease and mortality documented in seals and sea lions	[37,38]
Seals and sea lions (pinnipeds)	Lower respiratory tract (bronchi, bronchioles, lung)	Widespread on bronchial and bronchiolar epithelium, type 2 pneumocytes and alveolar macrophages	Variable α2,6-SA on airway epithelium and some alveolar cells	Favors efficient replication and severe pneumonia by HPAI H5Nx and other avian-like strains; co-expression of α2,6-SA may facilitate adaptation to mammalian transmission	[37,38]
Seals and sea lions (pinnipeds)	Intestine	α2,3-SA on intestinal epithelium and goblet cells	Limited data; α2,6-SA occasionally detected in subsets of enterocytes	Potential enteric infection and fecal shedding of AIVs, but respiratory route appears predominant during pinniped outbreaks	[153,154]
Seals and sea lions (pinnipeds)	CNS (brain, spinal cord)	α2,3-SA detected on neurons and glial cells in some species	α2,6-SA co-expressed in subsets of neurons and glia	Compatible with neurotropic infection; neurologic signs and encephalitis reported in some AIV-infected seals, suggesting CNS involvement once virus breaches blood–brain or olfactory barriers	[153,154,155]
Cetaceans (dolphins, porpoises, whales)	Upper and lower respiratory tract (blowhole, trachea, lung)	Evidence of α2,3-SA on respiratory epithelium and alveolar cells from lectin and glycan studies	α2,6-SA identified on subsets of airway epithelial and glandular cells	Mixed sialome suggests potential susceptibility to avian-like and adapted strains; serologic evidence of AIV exposure exists, but confirmed clinical outbreaks are less frequent than in pinnipeds	[156]
Cetaceans	Intestine, other organs	α2,3-SA present on intestinal epithelium and some glandular and endothelial structures	α2,6-SA variably detected in intestinal and hepatic tissues	Broad SA repertoire could support systemic replication of adapted AIVs; current epidemiological role remains poorly defined	[156]
Polar bears and other marine-associated carnivores	Respiratory tract, intestine	α2,3-SA on respiratory and intestinal epithelia (extrapolated from carnivore data and limited marine-carnivore studies)	α2,6-SA co-expressed in respiratory tissues in some species	Scavenging of infected birds and marine mammals may expose these predators to AIVs; receptor pattern suggests theoretical susceptibility to avian-like strains	[157,158]
Bats (Chiroptera, fruit bats and insectivorous bats)	Upper respiratory tract (nasal mucosa, larynx)	α2,3-SA detected on respiratory epithelium in several bat species	α2,6-SA also expressed on subsets of respiratory epithelial cells	Mixed α2,3/α2,6 sialome allows for binding of avian- and human-type HAs in vitro; some experimental infections demonstrate AIV replication in bat airways, but natural AIV infections appear rare	[49,159]
Bats (Chiroptera)	Lower respiratory tract (trachea, bronchi, lung)	Prominent α2,3-SA on lower airway epithelium and type II pneumocytes in some species	Variable α2,6-SA on bronchial epithelium and submucosal glands	Lower airway α2,3-SA may support replication of H5/H7 AIVs; co-expression of α2,6-SA in some bats raises concern about potential adaptation or reassortment with mammalian-tropic influenza viruses	[49,159]
Bats (Chiroptera)	Intestine	α2,3-SA on intestinal epithelium and Peyer’s patches–associated cells	α2,6-SA detected in subsets of enterocytes and glandular cells	Enteric replication of influenza viruses demonstrated experimentally in some bats (mostly bat-specific influenza A-like viruses); relevance for classical avian AIV ecology remains uncertain	[49,159]
Bats (Chiroptera)	CNS (brain, spinal cord)	α2,3-SA expressed on neurons and glia in several species	α2,6-SA co-expressed in defined neuronal/glial populations	CNS sialome compatible with potential neurotropism; experimental data for classical AIV are limited, but neuroinvasion is theoretically possible if systemic spread occurs	[49,159]

**Table 4:** Distribution of sialome receptors (α2,3-linked and α2,6-linked sialic acid [SA]) in tissues of different reptiles and relevance for avian influenza virus (AIV) potential infection.

Reptile Group	Tissue/System	α2,3-Linked SA Distribution	α2,6-Linked SA Distribution	Relevance for AIV/Notes	References
Chelonians (turtles, tortoises)	Oral and nasal mucosa, conjunctiva	Abundant on stratified and respiratory-type epithelia; strong *Maackia amurensis* lectin (MAL/MAL II) binding	Detected at lower density on some epithelial and glandular cells (*Sambucus nigra* agglutinin [SNA])	Prominent avian-type receptors on upper digestive/respiratory/ocular mucosa; theoretical entry sites for AIVs	[167]
Chelonians	Intestine, cloaca	Widespread on enterocytes and goblet cells along the intestinal tract	Variable and generally weaker than α2,3-SA; focal staining in some segments	Supports potential for enteric attachment of avian-type viruses; evidence based mainly on lectin histochemistry	[167]
Chelonians	Liver, kidney	α2,3-SA on bile duct epithelium, hepatocytes, and some renal tubules	Focal α2,6-SA on subsets of hepatocytes and renal tubular cells	Systemic tissues express both linkages; implications for systemic AIV spread remain speculative	[167]
Crocodilians (alligators, crocodiles)	Oral/upper respiratory mucosa	Predominantly α2,3-SA on mucosal and glandular epithelia	Low-to-moderate α2,6-SA in selected epithelial and glandular cells	Receptor pattern compatible with avian-type influenza binding; serologic evidence of influenza A virus exposure reported in farmed crocodilians	[168,169,170]
Crocodilians	Intestine, liver	α2,3-SA on intestinal epithelium and hepatic structures	Occasional α2,6-SA in hepatic and intestinal cells	Suggests potential for enteric infection by avian-type viruses; no direct AIV replication studies in these tissues	[168,169]
Lizards (iguanids, geckos, others)	Oral mucosa, gastrointestinal tract, skin, reproductive tract	α2,3-SA widely expressed on mucosal and some cutaneous and glandular epithelia	α2,6-SA variable; detected in subsets of gastrointestinal and reproductive epithelia	Mixed receptor profile could allow for binding of both avian- and human-type viruses; epidemiological role in AIV ecology remains unclear	[171]
Lizards	Gastrointestinal tract	α2,3-SA on enterocytes and goblet cells in the gastrointestinal tract	Patchy α2,6-SA on villus tips and some glands; species-dependent	Supports potential for enteric attachment of avian-type strains; data from lectin mapping only	[171]
Snakes (colubrids, vipers, and others)	Oral/esophageal mucosa, lung (faviform lung), intestine	α2,3-SA on mucosal and respiratory epithelia in several species	Generally low or focal α2,6-SA, sometimes confined to discrete glandular or neuronal populations	Presence of avian-type receptors in upper digestive/respiratory tract suggests potential susceptibility to AIVs; no confirmed natural AIV cases in snakes	[164]
Snakes	Intestine	Predominant α2,3-SA on enterocytes and goblet cells	Sparse α2,6-SA; if present, often segment-specific	Reinforces avian-type receptor bias in snake gut; implications for AIV infection remain theoretical	[164]
Various reptiles (chelonians, lizards)	CNS (brain, spinal cord)	Neuronal and glial α2,3-SA demonstrated by SA–specific lectins	α2,6-SA present in subsets of neurons and glia; species- and region-dependent pattern	CNS expresses both avian- and human-type linkages; influenza virus neurotropism in reptiles has not been documented	[171,172]

**Table 5:** Summary of sialome receptors (α2,3-linked and α2,6-linked SA) distribution in amphibians and fish and relevance for HPAIV infection.

Taxon/Group	Tissue/Surface	Evidence for α2,3-Linked SA	Evidence for α2,6-Linked SA	Relevance for HPAIV (H5/H7) Susceptibility	Key References
Amphibians (general)	–	Presence of Neu5Ac/Neu5Gc on glycoproteins and gangliosides widely documented; linkage-specific mapping scarce.	Same as α2,3-SA; both linkages suggested by lectin studies but not systematically mapped.	Amphibians share aquatic habitats with waterfowl and are exposed to AIVs in water; actual role as HPAIV hosts remains unproven; likely incidental contacts rather than reservoirs.	[173,174]
Amphibians—skin and mucus	Cutaneous epithelium, mucus glands	Sialylated O-glycans in skin and mucus; α2,3 linkage likely based on vertebrate mucosal patterns; direct linkage-specific data limited.	α2,6 also likely present in deeper epithelial/mucus glyco-conjugates; limited lectin data.	HPAI virions in water can bind sialylated mucus/skin; may allow for transient adsorption but productive infection not demonstrated. Potent cutaneous defenses (antimicrobial peptides, microbiota) probably restrict entry.	[174]
Amphibians—oral cavity/buccopharynx	Buccal and pharyngeal mucosa, salivary glands	Sialylated mucins and glycoproteins inferred from histochemical and biochemical works; pattern likely enriched in α2,3-SA like in other vertebrate oral mucosa; minimal direct mapping.	α2,6-SA probably co-expressed on select mucosal/ductal cells (lectin data from *Xenopus* and other anurans suggest both linkages).	Ingestion of AIV-contaminated water/prey could permit attachment of avian-like HA to α2,3-SA; no data showing replication or oral shedding of HPAIVs in amphibians.	[175]
Amphibians—intestinal tract	Small and large intestine mucosa, goblet cells	Intestinal mucus and epithelial glyco-conjugates contain SAs; limited lectin studies suggest predominance of α2,3-SA on luminal glycol-conjugates, by analogy with fish/birds.	α2,6-SA detected at lower intensity and/or more basally in vertebrate intestine; specific amphibian data sparse but likely present.	Compatible with avian-type intestinal tropism; however, no robust evidence of HPAIV replication or fecal shedding. Amphibians not recognized as enteric hosts for HPAIVs.	[174,176]
Amphibians—lungs/respiratory structures	Lungs (where present), glottis, upper airways	Sialylated epithelial glyco-conjugates inferred from general vertebrate patterns; no systematic α2,3-specific mapping performed with influenza probes.	α2,6-SA also likely present but unquantified.	For HPAIVs, a respiratory route analogous to birds/mammals not documented in amphibians. No field or convincing experimental evidence of significant lung infection by HPAIVs.	[177,178]
Amphibians—nervous tissue and eggs	Brain (gangliosides); oocyte/egg envelopes	Brain gangliosides with SAs (mostly α2,3-linked internal to galactose) are well documented in frogs. *Xenopus* egg envelopes contain sialylated glycoproteins.	α2,6-SA may occur on some glycoproteins but is less clearly characterized in these tissues.	HPAIV neuroinvasion in mammals is typically secondary and severe; for amphibians there is no evidence of CNS infection by HPAIVs. Eggs and embryos may contact contaminated water but no data on influenza infection.	[174]
Fish (teleosts—general)	–	Sialylated glyco-conjugates and gangliosides widespread; α2,3 commonly detected on mucosal and epithelial surfaces (gills, skin, gut).	α2,6 also present but often weaker and more cell-type restricted than α2,3 in mucosa.	Fish live in AIV-contaminated waters and clearly possess α2,3-/α2,6-SA, but no compelling evidence of productive HPAIV infection; considered environmental contacts/sinks rather than reservoirs.	[174,179]
Fish—gills	Gill lamellae and filaments, mucus	Multiple lectin histochemistry studies show strong MAL (α2,3) binding on gill epithelium and mucus in carps, salmonids, and sea basses.	SNA (α2,6) binding detectable but generally less intense and restricted to subsets of epithelial/submucosal cells.	Gills are a major interface with AIVs in water. α2,3-SA-rich surface can bind avian-like HA, but sustained HPAIV replication has not been demonstrated. Orthomyxovirus Infectious Salmon Anemia Virus (ISAV) (fish-specific) infects gills through 4-*O*-acetylated SA, not typical AIV receptors.	[180,181,182,183]
Fish—skin and mucus	Epidermis, mucus layer	Sialylated mucins abundant; α2,3-SA typically dominates on outer mucus/epithelium based on lectin and biochemical data.	α2,6 present but often lower; may localize to deeper epithelial layers or specific cell types.	Skin/mucus can adsorb AIVs from water via α2,3-SA, potentially reducing free virus titers and acting as a mechanical carrier. No evidence of cutaneous HPAIV infection or shedding.	[174,179]
Fish—intestinal tract	Intestinal epithelium, goblet cells, mucus	Strong α2,3-SA on brush border and mucus demonstrated in several teleosts (carp, trout) using MAL and other lectins; analogous to avian intestinal α2,3-SA dominance.	α2,6 present at lower levels; restricted to some epithelial or goblet cell subsets.	Theoretically permissive for avian-like HA binding upon ingestion of AIV-contaminated water or prey, but no consistent evidence of HPAIV replication or virus shedding; fish not considered enteric hosts for HPAIVs.	[111,180,182]
Fish—kidney/hematopoietic tissues	Pronephros (head kidney), mesonephros, blood cells	Sialylated glycoproteins on renal tubules and hematopoietic cells; α2,3 frequently detected by lectins.	α2,6 also present on some renal and immune cell populations.	These are secondary/hematopoietic tissues; influenza virus primary infection should be systemic. No evidence for systemic HPAIV infection in fish.	[174,179]
Fish—CNS	Brain, spinal cord (gangliosides)	Complex sialylated gangliosides (often α2,3 on internal galactose) well characterized in trout and other teleosts.	External terminal α2,6 not well characterized; likely present on some glycoproteins.	Influenza neurotropism in warm-blooded hosts is secondary; no reliable reports of HPAIV neuro-infection in fish.	[174,184]
Fish—comparison: Infectious Salmon Anemia Virus (ISAV)	Gills, vascular endothelium	ISAV HA-like protein binds 4-O-acetylated SAs (a modified α2,3-SA) on gill and endothelial cells.	Not dependent on classical α2,6-SA for entry.	Demonstrates that fish are Orthomyxovirus hosts but receptor specificity is distinct from HPAIV; supports the idea that simple α2,3/α2,6 presence is insufficient to predict AIV susceptibility—fine structure and modification of SA are critical.	[181,185]

## Data Availability

No new data were created or analyzed in this study. Data sharing is not applicable to this article.
